# Can NMR-HetCA be a Reliable Prediction Tool for the Direct Identification of Bioactive Substances in Complex Mixtures?

**DOI:** 10.1021/acs.analchem.4c05080

**Published:** 2024-12-06

**Authors:** Vaios Amountzias, Antigoni Cheilari, Argyro Vontzalidou, Dimitra Benaki, Evagelos Gikas, Nektarios Aligiannis

**Affiliations:** 1 Department of Pharmacognosy and Natural Products Chemistry, Faculty of Pharmacy, National and Kapodistrian University of Athens, Panepistimiopolis Zografou, Athens 15771, Greece; 2 Department of Pharmaceutical Chemistry, Faculty of Pharmacy, National and Kapodistrian University of Athens, Panepistimiopolis Zografou, Athens 15771, Greece; 3 Department of Analytical Chemistry, Faculty of Chemistry, National and Kapodistrian University of Athens, Panepistimiopolis Zografou, Athens 15771, Greece

## Abstract

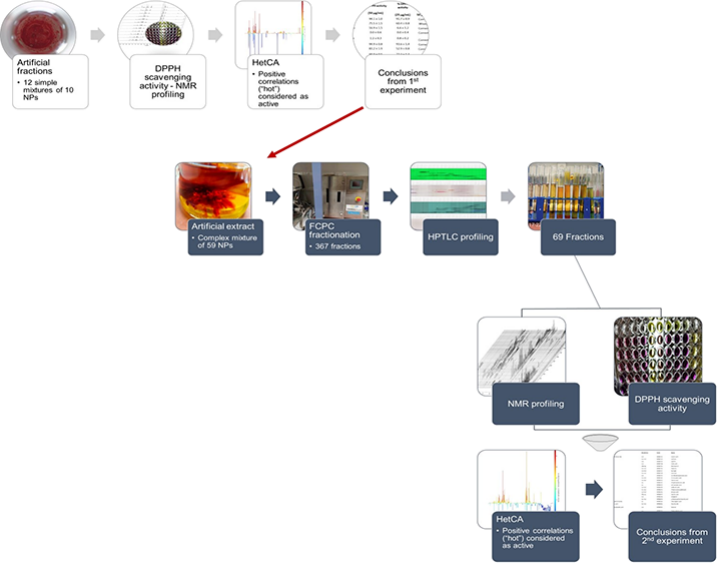

Conventional isolation methods in natural products chemistry are time-consuming and costly and often result in the isolation of moderately active compounds or the detection of already known natural products (NPs). A fast and cost-effective way to identify bioactive metabolites in plant extracts prior to isolation has been developed based on the nuclear magnetic resonance (NMR)-heterocovariance approach (NMR-HetCA). In order to evaluate in depth the application of this chemometrics-based drug discovery methodology, simple mixtures of 10 standard NPs simulating a fast centrifugal partition chromatography (FCPC) fractionation (artificial fractions, ArtFrcts), as well as a more complex mixture of 59 natural standard substances simulating a crude plant extract (artificial extract, ArtExtr), were prepared. FCPC was employed for the fractionation of the ArtExtr, while the inhibitory activity of all fractions against DPPH was evaluated, and their chemical profile was recorded using NMR spectroscopy. Spectral information was processed in the MATLAB environment, and statistical approaches, including HetCA and statistical total correlation spectroscopy (STOCSY), were applied to identify bioactive compounds. Total heterocovariance plots (pseudospectra) facilitated the detection of highly correlated metabolites and led to the direct identification of 52.6% of the active compounds. The success in identifying the ArtExtr bioactive substances increased to 63.2% when spectral alignment was implemented. HetCA incorporates chromatographic (fractionation), spectroscopic (NMR profiling), and bioactivity results along with advanced chemometrics and could be established as a method of choice for the rapid and effective identification of bioactive NPs in plant extracts prior to isolation.

## Introduction

Conventional methods used for the isolation and identification of bioactive constituents of crude extracts in NPs chemistry are time-consuming, costly, and often result in the reisolation and reidentification of known molecules or the detection of moderately and/or nonactive ones.^1,2^

The need for the fast identification of secondary metabolites in extracts prior to their isolation has led to the development of powerful analytical methods that facilitate high-throughput screening and dereplication strategies.^3−6^ Two important tools are the STOCSY^7^ and the statistical heterospectroscopy (SHY),^8^ operating in the MATLAB environment. STOCSY benefits from the multicollinearity observed among the intensity variables within a collection of spectra, e.g., ^1^H NMR spectra. It creates a pseudo-two-dimensional NMR spectrum, illustrating the correlations among the intensity levels of different peaks throughout the entire sample based on the Pearson correlation coefficient.^7,9^ SHY functions by examining the inherent covariance among signal intensities within the same or associated molecules, as determined by measurements using different techniques (e.g., NMR and MS) across sets of samples.^8^ A similar approach to STOCSY, using semiselective or selective TOCSY, was proposed by Sandusky and Raftery^10,11^ for the detection of minor compounds in honey and urine. STOCSY and/or SHY have been used for the identification of metabolites in biofluids and plant extracts,^12−15^ while Borges et al.^16^ created a free-to-use pipeline called Data Fusion-based Discovery (DAFdiscovery) using these algorithms in the Python environment. Similar approaches to SHY are the 3D cross-correlation (3DCC), used for the characterization of complex glycan mixtures by integration of ^1^H NMR and LC-MS,^17−19^ as well as the calculation of Pearson's correlations using Excel (Microsoft, Redmond, WA, USA).^20^ Other approaches for the dereplication of secondary metabolites in extracts include the combination of separate dereplication processes carried out by ^1^H NMR and LC-MS using databases and the comparison of the predicted ^1^H NMR spectra of the identified compounds with the experimental ones.^21−23^ In addition to the dereplication of secondary metabolites with the help of MS, a few studies have utilized NMR databases^24−27^ regarding ^13^C^28−31^ or 2D^32−34^ experiments.

The need to identify bioactive compounds in extracts necessitated the correlation of the spectral data with the bioactivity. In pursuit of this objective, several studies have employed chemometrics, notably utilizing orthogonal partial least squares to latent structures (OPLS) modeling.^35,36^

A more recent methodology toward detecting the bioactive components of a mixture prior to their isolation is HetCA that was developed in our lab.^37^ HetCA implements a modified SHY algorithm and uses the *crosscov* and *corr* functions to calculate the covariance and Pearson's correlation coefficient between the ^1^H NMR spectra and corresponding biological activity. Among the different programming languages, the MATLAB environment is used. The result is a plot resembling a ^1^H NMR spectrum (pseudospectrum), where the covariance and correlation coefficient between NMR resonances and activity values are visualized. There have been a few publications using this approach to correlate bioactivity with the spectral data of secondary metabolites.^38−40^

In our group, chemometrics-based drug discovery methodologies have been successfully applied in the past for the detection of secondary metabolites in plant extracts that were active against tyrosinase, acetylcholinesterase, and hyaluronidase.^37,41−43^ In these studies, biological activity results were statistically correlated with NMR and/or high-performance thin layer chromatography (HPTLC) profiles, while for the detection of bioactive substances, descriptive and/or multivariate statistics were implemented, respectively. In the case of NMR, HetCA was proved to be powerful in the detection of bioactive compounds, including minor ones, prior to isolation, such as 2,4,3′-trihydroxydihydrostilbene in *Morus alba*(37) and 1,2,3,4,6-penta-*O*-galloyl-β-d-glucopyranose (PPG) in *Paeonia parnassica*.^43^ Nevertheless, a limited number of substances have been detected or identified relative to those present in an extract. The underlying cause of this observation can be due to three main reasons as follows: (a) issues of the chromatographic process, (b) choice of data processing methods, and (c) the difference in substance content (major and minor compounds) and their concentration variance within the extract and fractions. Moreover, the result of falsely positive detections arose questions about whether NMR-HetCA can detect all the bioactive compounds in the sample. In any case, the encouraging results of these works prompted us to conduct a thorough evaluation of this chemometrics-based methodology, both to determine its success rate and to engage in process optimization through the identification of factors that contribute to the inadequate identification of bioactive ingredients.

As a first step, the fractionation of a plant extract by FCPC was simulated, overcoming issues possibly caused by the chromatographic process by using 10 standard substances to prepare 12 ArtFrcts. DPPH assay was used to test the scavenging activity of the ArtFrcts due to its reproducibility, simplicity, and quantitative results, as well as the availability of literature data and the low cost of the assay. Furthermore, the additive activity of the compounds is mostly observed. NMR-HetCA analysis was applied to all of the spectral and bioactivity information (total HetCA) to evaluate the algorithm performance under controlled conditions. As a second step, a crude plant extract was approximated by preparing a complex mixture of 59 standard substances (ArtExtr) and the chromatographic fractionation step was involved in the evaluation of HetCA performance. One of the most important steps in the effective detection of bioactive secondary metabolites through HetCA is fractionation of the initial mixture. An efficient fractionation ensures sufficient variance of the concentration of the compounds in several fractions. This concentration variance, in correlation with the variation of the fractions’ activity through HetCA, can result in the detection of the bioactive compounds. Among the chromatographic techniques, FCPC was selected for the fractionation of the ArtExtr since it is advantageous due to its high loading capacity, the lack of solid stationary phase that could withhold basic compounds, the high sample recovery, the broad elution range, the easy change between normal (NP) and reverse (RP) phase, the wide range of solvents that can be used, and the high separation capability with the selection of a proper solvent system.^44,45^ The fractions resulting from FCPC were profiled by HPTLC and ^1^H NMR, and their antiradical activity was evaluated (Figure S1). Applying total HetCA analysis in a complex artificial mixture of known compounds bearing various chemical properties, we aimed to explore possible challenges during plant extract analysis through a systematic and thorough investigation. Furthermore, this study significantly improves the understanding of the application of STOCSY, SHY, and HetCA methodologies in complex mixtures analysis, since they share the same principle of method, highlighting their pros and cons by utilizing mixtures of standard compounds.

## Experimental Section

### Solvents and Reagents

All solvents were of analytical grade and were purchased from Merck (Merck, Darmstadt, Germany), while 2,2-diphenyl-1-picrylhydrazyl (DPPH) was purchased from Sigma-Aldrich (Sigma-Aldrich, Steinheim, Germany). Water was produced by a LaboStar PRO TWF system (Evoqua Water Technologies, Pittsburgh, USA). For the standard compounds, see the Supporting Information.

### Sample Preparation

#### ArtFrcts Preparation

Ten standard compounds listed in Table S1, in various concentrations, were used for the preparation of 12 ArtFrcts (Figure S2). The samples were assayed for *in vitro* antiradical assay evaluation and were analyzed by NMR-HetCA.

#### ArtExtr Preparation

A mixture (ArtExtr) composed of 59 standard compounds (Figure S2 and Table S2) was prepared with 50 mg of each compound diluted in 50 mL of MeOH. Mole fractions of all compounds were used in order to simplify the concentration and activity covariance study (Table S2).

#### FCPC

The fractionation of the ArtExtr was performed by FCPC (FCPC KROMATON, France) with a 1000 mL column and adjustable rotation of 650–1700 rpm, equipped with a Gilson PLC 2250 pump and a fraction collector compact system (Gilson Incorporated, Middleton, USA). The ArtExtr was fractionated using a step-gradient elution–extrusion method consisting of *n*-Hept, EtOAc, *n*-BuOH, MeOH, and H_2_O in ascending mode (See Supporting Information, Table S3). The resulting 69 pooled fractions were filtered and forwarded for NMR-HetCA, HPTLC profiling, and *in vitro* antiradical assay evaluation.

#### Evaluation of Free Radical Scavenging Activity by DPPH Assay

The DPPH assay was used for the biological screening for the standard substances, ArtFrcts and ArtExtr fractions, as previously described by Lee et al.^46^ with minor modifications (See Supporting Information). The absorbance was measured at 517 nm, using a Tecan Infinite M1000 PRO microplate reader (Tecan GmbH, Grödig/Salzburg, Austria), while the system was operated under Tecan i-control v.1.11. All evaluations were performed in triplicate, while gallic acid was used as the positive control (IC_50_ = 30.2 μM). The *in vitro* DPPH inhibition assay for the ArtFrcts, as well as for the pure substances, was performed at 50 and 25 μg/mL. In the case of standard substances comprising the ArtExtr, IC_50_ values were calculated for the most active ones. Regarding the ArtExtr FCPC fractions, the evaluation took place at a final concentration of 75 μg/mL in the well.

#### HPTLC

For the chemical profiling of the ArtExtr fractions, the filtered samples dissolved in MeOH were applied on HPTLC plates by using an automatic TLC Sampler 4 (ATS-4, CAMAG, Muttenz, Switzerland). The chromatographic separation was performed in an Automatic Developing Chamber 2 (ADC 2), while the documentation was carried out in CAMAG Visualizer 2. The system was operated under the VisionCats 3.0 software (CAMAG) (See Supporting Information).

#### NMR Spectroscopy and Data Pretreatment

For the ^1^H NMR experiment, the samples were dissolved in methanol-*d*_4_ containing tetramethylsilane (TMS) as a reference (Euriso-Top, Saint-Aubin, France) at a concentration level of 10 mg/mL for the unfiltered ArtFrct samples and 3 mg/mL for the filtered ArtExtr FCPC samples. After sonication (5 min) in an Ultra Sonic bath (Elma Schmidbauer GmbH, Singen, Germany), 650 μL was transferred to 5 mm NMR tubes (LabScape, Bruker, Germany). The ^1^H NMR spectra were acquired at 298 K ± 0.1, after a 5 min resting period for temperature stabilization, on a Bruker Avance III 600 MHz NMR spectrometer equipped with a 5 mm PABBI 1H/D-BB inverse detection probe. Experiments were performed in automation mode using a BACS-60 sample changer operated by IconNMR. Data acquisition and processing were done with Bruker TopSpin 3.6. Profiling ^1^H NMR spectra were acquired using the water suppression 1D NOESY pulse program with the following settings: relaxation delay (d1) = 6 s, acquisition time = 2.73 s, FID (free induction decay) data points = 64 k, spectral width = 20 ppm, and number of scans = 128. The transmitter offset was set manually in order to achieve the optimal suppression of the residual water signal. FIDs were multiplied by an exponential weighting function corresponding to a line broadening of 0.3 Hz prior to Fourier transformation. Automated processing was carried out for phase correction and baseline correction. Chemical shift values were referenced to the residual methanol signal (3.31 ppm). ^1^H NMR spectral alignment was based on a segment-wise peak alignment and was performed in pairs, with the last one set as the “active” spectrum for the alignment of the next spectrum using MestRe Nova 14.2.1 (Mestrelab Research, Santiago de Compostela, Spain) using the implemented cross-correlation algorithm. The first derivative was used, while the missing values filling method was linear.

#### NMR-HetCA Approach

Regions excluded from the integration of the ^1^H NMR spectra were the residual methanol-*d*_4_ signal (3.29–3.36 ppm) and the water peak (4.76–4.82 ppm). NMR spectra were processed in the MATLAB environment (bucketing of spectra and correlation with DPPH radical scavenging activity) through HetCA as previously described.^37^ The covariance and correlation between NMR resonances and activity values were visualized through the generated NMR pseudospectra, i.e., the HetCA plots. Each point of the HetCA plots depicted the covariance, where positive or negative peaks indicated positive or negative covariance values, respectively. They were additionally color-coded according to the respective correlation coefficients, ranging from blue for those that show low correlation to deep red for those that show high correlation. Besides HetCA plots, structural identification of the compounds in the pseudospectra was further accomplished by applying STOCSY on selected peaks and by comparing with the ^1^H NMR spectra of the standard substances.

## Results and Discussion

### Detection of Bioactive Compounds in the ArtFrcts with NMR-Total HetCA

The first step in the evaluation of HetCA performance was its application on a set of simple mixtures (ArtFrcts) with known composition in order to simulate an FCPC fractionation of low complexity. Hence, 12 ArtFrcts were prepared by combining 10 standard compounds in sequentially variable concentrations (Table S1). The standard compound set was selected to include strong (e.g., quercetin, Qtn) DPPH scavengers, as well as inactive substances (e.g., naringenin, Nr). The DPPH inhibition assay for the ArtFrcts was performed at two concentrations, 50 and 25 μg/mL (Figure S3), due to the fact that most of the ArtFrcts (7) had reached a plateau at 50 μg/mL (>80%). When bioactivity reached plateau values, the linear concentration–activity relationship did not apply, so there is no reliability in the measurement. At 25 μg/mL, the bioactivity variance was low but the results were reliable. Thus, total HetCA was applied using the activity values from 25 μg/mL and the results are shown in Table S4. The pseudospectrum (Figure S4) generated from total HetCA had both positive and negative peaks indicative of active and nonactive contributions, respectively. Accordingly, a color code was used and resonances exhibiting high correlation were colored in deep red, while those of low correlation were in blue.

The results of the experiment showed that all active compounds were correctly predicted (Figure S4), except ferulic acid (Fr) due to its limited solubility during the NMR sample preparation. In general, NMR spectroscopy requires samples of higher concentration than biological assays (1 order of magnitude or more). Therefore, the reduced solubility of Fr in the NMR samples resulted in saturated solutions with lack of accuracy and variance of its concentration in the respective ArtFrcts. On the contrary, Fr was fully dissolved in the corresponding bioassay samples. The reduced solubility of Fr in the NMR samples caused an inconsistency between the NMR data and the biological response and resulted in false correlation results. The low bioactivity variance did not affect the results of this study, probably due to the simplicity in the composition of the ArtFrcts.

### Detection of Bioactive Compounds in the ArtExtr with NMR-Total HetCA

#### ArtExtr Preparation and Fractionation

As a next step in the thorough evaluation of NMR-HetCA performance, this methodology was applied on a more complex mixture (ArtExtr) composed of 59 standard substances. The constituents of the ArtExtr were selected to cover (a) a wide range of polarity, from nonpolar (e.g., oleanolic acid, **14**; Table S2) to polar (e.g., sucrose, **25**) compounds, and (b) different chemical categories and (c) various scavenging activities against DPPH, including strong inhibitors (e.g., quercetin, **03**), as well as inactive compounds (e.g., palmitic acid, **08**), in order to simulate a plant extract. The *in vitro* evaluation of their scavenging activity at 100 μg/mL showed that 39 out of 59 compounds were inactive (0.0–50.0% DPPH inhibition), and 20 possess significant antiradical activity (50.1–100.0% DPPH inhibition) (Table S2). In order to establish the selectivity of total HetCA and the possibility of the identification of similar compounds, substances with similar ^1^H NMR spectra were also selected (e.g., 3,5-dihydroxybenzoic acid and protocatechic acid, **39** and **53**, respectively), possessing different activities against DPPH free radicals. Compounds that have acidic or basic properties and often exhibit minor chemical shift variations in ^1^H NMR related to their concentration^47^ were also selected. This fact increases the degree of difficulty regarding the treatment of the mixture and the management of the resulting fractions, but helps to draw conclusions about the most effective way to manage a plant extract, which contains compounds with the aforementioned properties (e.g., phenolic acids, triterpenic acids, and alkaloids).

The amount of each standard compound used for the preparation of the ArtExtr was 50 mg, while the mmoles and the mole fraction of each substance in the mixture are shown in Table S2. A part of the ArtExtr (1.2 g) was chromatographed by FCPC, resulting in 367 fractions (20 mL/fraction). These were pooled into 69 fractions aiming for the optimal variance of the compounds based on their TLC profiles and filtered in order to avoid precipitation.

#### Fraction Profiling and Evaluation of Their Activity

Subsequently, the chemical content of the pooled fractions was evaluated by NP and RP HPTLC (Figure S5) and the fractionation was deemed as adequate since most of the compounds have been distributed in several fractions.

**Figure 1:**
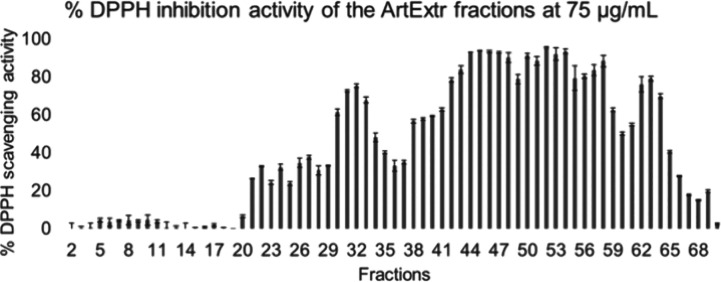
%DPPH inhibition of all the ArtExtr fractions at 75 μg/mL. Inhibition is expressed as mean ± SD (*N* = 3).

The ArtExtr FCPC fractions were evaluated at a final concentration of 75 μg/mL (Figure 1). At higher concentrations tested (200 μg/mL, data not shown), the variation of the activity was limited because the inhibition of DPPH was higher than 80% for a significant number of fractions (36), prohibiting the identification of bioactive components, as aforementioned.

#### Preliminary NMR Spectra Evaluation

The pooled ArtExtr fractions were dissolved in methanol-*d*_4_ at a concentration level of 3 mg/mL and analyzed by ^1^H NMR. The signals of seven substances were not observed in the ^1^H NMR spectra. These compounds were **01** (galanthamine hydrobromide), **09** (reserpine), **12** (ephedrine), **13** (harmine), **20** (oxytetracycline hydrochloride), **31** (tannic acid), and **40** (quinic acid) (Figure S6). Among the aforementioned substances, compounds **01**, **09**, **12**, **13**, and **20** belong to the category of alkaloids and are characterized by limited solubility in organic solvents. Therefore, we concluded that the formation of a precipitate occurred during the introduction of the initial mixture into the separation column, which contained water as the stationary phase (Table S3). This, coupled with the high water solubility of certain compounds, led to the elution of some substances (e.g., **13**) in the extrusion fraction at the end of the separation process. In general, for the fractionation of extracts containing alkaloids, more polar biphasic systems are needed, or, more preferably, pH-zone-refining FCPC.^48^ Moreover, the overlap of NMR peaks belonging to several compounds of the mixture resulted in difficulty in the detection of some compounds in the ^1^H NMR spectra (e.g., **40**). Finally, **31** was not detected in the NMR spectra of the fractions, probably due to its very small mole fraction (0.24%; Table S2) in the initial mixture and the fact that it was a mixture of polygalloyl glucoses or polygalloyl quinic acid esters.

In the stack plot of the ArtExtr fractions’ ^1^H NMR spectra, misalignment was observed in many of the peaks that corresponded to the same compounds between consecutive fractions. Such an example is presented in Figure S7. The high sensitivity of ^1^H nuclei to their chemical environment results in the resonance dispersion, allowing structure identification. On the other hand, in the absence of buffering conditions (in organic solvents), like in this study, the most sensitive ^1^H display various chemical shift variations, hindering the statistical analysis of the spectroscopic data. Several alignment tools have been developed^49,50^ underlining the importance of this procedure. In the current work, alignment was achieved through MestRe Nova 14.2.1 software based on a segment-wise peak alignment in pairs, with the last spectrum being the “active” one for the alignment of the next. This method was preferred to the alignment of the spectra altogether since different compounds can have peak signals at similar δ_H_, while being located in different fractions, so the integration as well as the correlation process would be incorrect.

#### NMR-HetCA Approach

**Figure 2:**
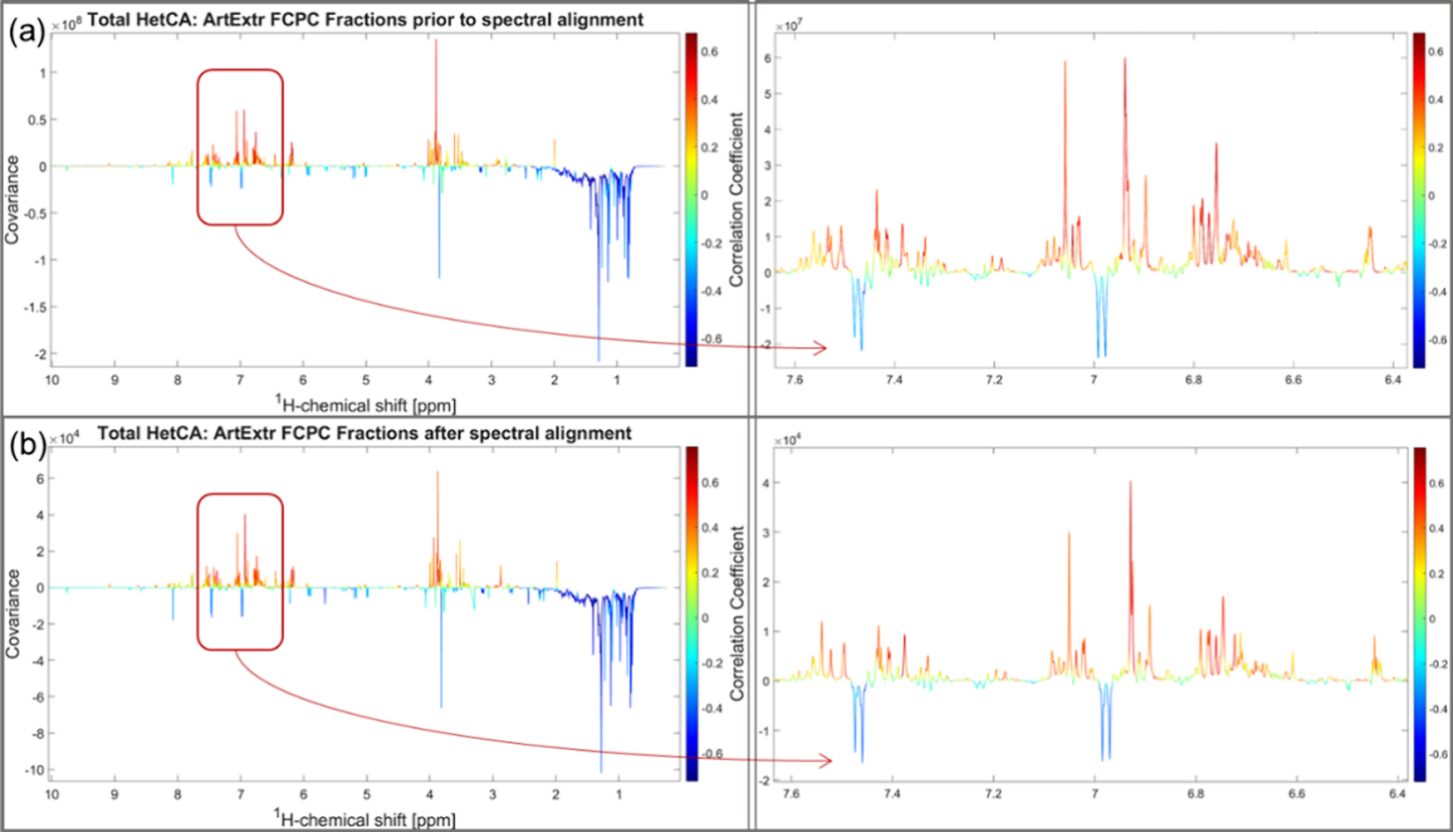
Total HetCA plots and zoomed areas (10.00−0.00 and 7.60–6.40 ppm, respectively) resulted from the covariance of the ArtExtr fractions’ NMR data with their corresponding DPPH scavenging activity (a) prior to spectral alignment and (b) after spectral alignment. The left *Y* axis of each HetCA plot denotes the covariance, the right *Y* axis represents the correlation coefficient, and the *X* axis indicates the ^1^H-chemical shift (ppm).

The application of total HetCA in the ^1^H NMR spectra before the application of the alignment resulted in the identification of 30 of the 52 compounds included in the study with the help of the STOCSY algorithm and the comparison with the ^1^H NMR spectra of the standard compounds. Total HetCA approach predicted correctly 21 of them (70.0%; Figure 2a), while there were 9 false positives and no false negatives. This method helped us to predict correctly 10 out of 19 bioactive compounds (52.6%) that were included in the study. The results are summarized in Table S5.

When total HetCA was applied to the aligned ^1^H NMR spectra, the acquired pseudospectrum was of higher analysis (Figure 2b) and the results were improved: 33 of the 52 compounds included in the study were identified, while 27 of them were predicted correctly (81.8%), and 5 false positives and 1 false negative were found (Table S6). This procedure contributed to the correct prediction of 12 out of 19 bioactive compounds (63.2%; Figures S8 and S9) that were included in the study (Figure S10). In general, 19 substances were not detected in the total HetCA plot and therefore were not characterized in terms of their correlation to the activity. For six compounds, a significant deviation was observed between the activity predicted by total HetCA and the activity determined by the *in vitro* assay. In order to further analyze the results, we created a table with the absolute integration value of a selected NMR peak for each of the detected compounds (integration table).

No deconvolution was applied, and hence, NMR signals without any overlapping were selected, besides the case of compound **10**. The approximate percentage content per fraction of each compound predicted as active is shown in Table S7.

#### Underestimation of Activity

The case of underestimation of activity is that of **38** (catechol). More precisely, it exhibits an *in vitro* IC_50_ of 48.2 μM against DPPH, so it is considered as one of the very active compounds present in the ArtExtr, while its characteristic peaks show a correlation close to zero in the total HetCA plot (Figure S11), suggesting an insignificant contribution to the activity. For a thorough and detailed investigation, the concentration of **38** and that of other compounds present in the respective fractions is examined. The concentration variance of compounds **04**, **16**, **38,** and **56** (kaempferol, hesperetin, catechol, and baicalein, respectively) are shown in Figure S12. The variance in the concentration of **38** and the activity of the respective fractions are in parallel for Fr25–30, where it is the main active component, while a significant deviation is observed in Fr31–33 (Figure S12). The activity variation of Fr25–29 is low, as are its absolute values (between 23.8 and 37.6%). The low activity of Fr25–29 indicates that the concentration of **38** is not sufficient to cause an adequate DPPH inhibition for **38** to be predicted as an active compound in total HetCA. In fractions Fr30–33, the activity increases by a large amount and ranges between 61.6 and 75.3%, in parallel with the ascending concentration of **56** (baicalein, IC_50_ = 26.3 μM), justifying its correct prediction through total HetCA, since it seems that this is the main compound causing the inhibition of DPPH in these fractions. On the other hand, the observed constant decrease of the concentration of **38** in these fractions rules out this compound from a positive contribution.

To further study the results, the complexity of the total HetCA plot was decreased by applying the methodology in groups of five consecutive spectra (partial HetCA) and the pseudospectra obtained were studied (Figure S13). The plots concerning Fr25–30 (Figure S13a,b,c) showed that the signals corresponding to **38** were highly correlated, showing that it contributed decisively to the activity, contrary to Fr31–33 (Figure S13d,e) where its signals were poorly correlated. The results were in perfect agreement with the aforementioned observations regarding the covariance of concentration and activity.

#### Compounds Not Detected in Total HetCA

As mentioned above, 19 compounds were not detected in the total HetCA plot due to their low concentration in the fractions and/or the overlap of their peaks. In order to explain the results, the example of **04** (kaempferol) was used. Compound **04** exhibited an *in vitro* IC_50_ of 68.1 μM against DPPH, and therefore, it was considered as one of the very active compounds present in the ArtExtr. Its concentration in the corresponding fractions was very low, while it seemed to contribute little only to fraction Fr24, although the activity of this fraction remained at low levels (32.4%; Figure S12). The activity of **04** was overshadowed by the presence of the more concentrated compounds **38** (catechol) and, more notably, **56** (baicalein) in the respective fractions (Fr24–37; Figure S12). This affected the covariance between **04** and the activity, rendering it very low. This, along with the fact that **04**’s ^1^H NMR peaks were in the same δ_H_ as those of other compounds with higher covariance with the activity, made the detection of **04** in the total HetCA very difficult.

#### Overestimation of Activity

As aforementioned, five compounds were overestimated in terms of activity in the total HetCA. The construction of the table with the integrals of the observed components of certain fractions aided the conduction of a very important observation: in most cases, the compounds with overestimated activity from the total HetCA approach exhibited high covariance in concentration with active compounds. For example, the concentration variance of the inactive compounds **05** (phlorizin) and **59** (colchicine) was analogous to that of the active compound **02** (quercitrin) (correlation was 0.95 and 0.94, respectively; Figures S14 and S15). Similarly, the concentration variation of the inactive compounds **32** (caffeine) and **39** (3,5-dihydroxybenzoic acid) resembled that of the active compounds **56** (baicalein) and **30** (ellagic acid) (correlation was 0.73 and 0.86, respectively; Figures S16 and S17). On the other hand, the wide distribution of **17** (nicotinic acid) in a large number of fractions (signals observed in fractions Fr30–60) resulted in its reduced amount per fraction (Figure S18). Its very low concentration, dispersed in numerous fractions, resulted in it not being one of the major compounds in terms of concentration in any of the fractions. Nevertheless, the continuous presence of its signals in the ^1^H NMR resulted in the incorrect prediction regarding to its contribution to the activity, rendering it a false positive.

#### Application of Sequential Partial HetCA

In the total HetCA plot, all spectral and bioactivity information were combined in a single pseudospectrum. The absence of some compound peaks in the total HetCA plot prompted us to apply HetCA in a smaller number of fractions. For fractions Fr20–70, exhibiting considerable activity values, HetCA was applied in consecutive sets of five fractions (partial HetCA), resulting in 47 pseudospectra. This approach led to the additional detection and correct prediction of (a) a bioactive compound (chlorogenic acid; **50**) and (b) nine inactive compounds (Table S8), all not detected in the total HetCA plot. However, missing the entire information led to unreliable results from partial HetCA. Overall, there were six false negative predictions, six false positives, and eight ambiguous results, where the same compounds seemed to contribute both greatly and not at all to the activity depending on the selected set of spectra (Table S8). These results seemed to be affected only by the covariance of the compound concentration with the activity, but not by the slope of the bioactivity itself, either positive or negative. Although the partial HetCA aided in the detection of additional active compounds in our previous studies, its application to the current controlled set exposed the limitations of this method. The reduction of the prediction accuracy led to unreliable results, which were likely attributed to the presence of multiple active compounds within the same fractions. These results were not taken into account regarding the final results.

## Conclusions

In this study, HetCA methodology’s possibilities and limitations were evaluated under controlled conditions using the ArtFrcts and the ArtExtr fractions. A certain variety of standard compounds was selected for the preparation of artificial mixtures aiming to cover several issues that occur during complex mixture analysis including sample preparation and use of various chromatographic, spectroscopic, and/or bioactivity techniques. This approach facilitated the detection and identification of the majority of bioactive compounds in these mixtures without the need for their isolation, offering solutions to the challenges discussed below.

Special care is required to include all of the components of the respective mixture in the study or to identify and record the components that have been removed during the chromatographic fractionation procedures and/or during the NMR process and biological assays. Due to the structural complexity and the wide polarity range of the components, precipitation is often observed during the preparation of the initial sample, resulting in the loss of a part of the extract from the chromatographic separation, and consequently from the study. Moreover, precipitation sometimes occurs during the preparation of the samples, both for NMR experiments and for *in vitro* biological assays, resulting to an underestimation of some compounds’ concentration and/or activity. Therefore, it is considered reasonable to study the physicochemical properties of the components of a mixture (e.g., plant extracts containing alkaloids) in order to optimally choose biphasic solvent systems for the fractionation by FCPC and to improve the processing protocols of the samples under study in order to avoid the aforementioned phenomena. All techniques should be carried out with the same set of prepared samples to avoid different concentration issues between them.

The components’ wide distribution in the fractions resulting from the chromatographic fractionation is crucial for the successful application of the HetCA, which can be achieved by FCPC. However, the choice of biphasic solvent systems, as well as the chromatographic method, is decisive for achieving a satisfactory distribution of the substances. The profiling of the chemical content of the produced fractions, both by chromatographic (HPTLC) and spectroscopic (NMR) techniques, is considered appropriate to evaluate the distribution of the substances before applying HetCA. The preparation of the samples, the acquisition of ^1^H NMR spectra, and the evaluation of the biological properties of the fractions need to be carried out by the same protocols, respectively, in order to minimize the influence of the aforementioned procedures on the results of the study.

The absence of buffering conditions when using organic solvents can result in minor chemical shift variations in the ^1^H NMR spectra due to the coexistence of acidic and basic compounds. Therefore, spectra alignment is crucial for more reliable results, since compounds with misalignment issues will be hindered from the generated HetCA plots. Moreover, the HetCA plots are more well-defined and easier to interpret, leading to more reliable results. In total HetCA, all fractions are considered, the whole range of biological activity values is included, and the results are more reliable, compared to pseudospectra generated from small groups of spectra.

As discussed, NMR-HetCA can give some misleading results (false-negative and false-positive identification of bioactives). The main cause for false negative results is the coelution of active compounds at low concentration with more active compounds at a higher concentration. On the other hand, false positive results can be produced by a comparable distribution of active and nonactive substances in the same fractions, phenomena of cumulative and/or synergistic activity of the coeluted substances, and a low variation in the bioactivity of the fractions. Nevertheless, the combination of selected procedures in HetCA methodology, such as FCPC fractionation and NMR spectral data correlation with bioactivity, facilitates the detection and identification of the majority of bioactive compounds and reduces the laboratory time needed to study a plant extract, since reisolation of known compounds is omitted. Besides time, the cost of a phytochemical study is decreased as well by reducing the volume of organic solvents needed. Furthermore, the detection of minor metabolites, which would otherwise be very difficult with conventional methods, can be achieved, making NMR-HetCA a competent tool in natural product research.

Overall, the HetCA approach can be used as a method of choice for the detection and identification of the majority of the active substances in a complex mixture prior to their isolation, provided that special care is taken in order to minimize the aforementioned reasons that cause incorrect predictions. Further investigation is needed for more complex bioactivity essays (e.g., enzymes), since the synergism and antagonism of the mixture ingredients are common.

## Supporting Information Available

Additional experimental details for HPTLC, FCPC, and DPPH assay, details on solvents and reagents, figures of total and partial HetCA plots, STOCSY plots, concentration–variance plots, tables of results regarding DPPH and HetCA methodologies, graphical representations of experimental design and results (PDF)
