# Supplementary material for: Can NMR-HetCA be a Reliable Prediction Tool for the Direct Identification of Bioactive Substances in Complex Mixtures?
Source: Anal Chem. 2024 Dec 6;96(50):20090–7. doi: 10.1021/acs.analchem.4c05080 (PMC11656414; doi:10.1021/acs.analchem.4c05080)
Supplement: Supplementary file 1 — ac4c05080_si_001.pdf [file ac4c05080_si_001.pdf]

Supporting Information

Can NMR-HetCA be a reliable prediction tool for the direct identification of bioactive substances in complex mixtures?

Vaios Amountzias[1], Antigoni Cheilari[1,*], Argyro Vontzalidou[1], Dimitra Benaki[2], Evagelos Gikas[3] and Nektarios Aligiannis[1,]

[1]Department of Pharmacognosy and Natural Products Chemistry, Faculty of Pharmacy, National and Kapodistrian University of Athens, Panepistimiopolis Zografou, 15771, Athens, Greece;

[2]Department of Pharmaceutical Chemistry, Faculty of Pharmacy, National and Kapodistrian University of Athens, Panepistimiopolis Zografou, 15771, Athens, Greece;

[3]Department of Analytical Chemistry, Faculty of Chemistry, National and Kapodistrian University of Athens, Panepistimiopolis Zografou, 15771, Athens, Greece

ABSTRACT: Conventional isolation methods in natural products chemistry are time-consuming and costly and often result in the isolation of moderately active compounds, or the detection of already known natural products (NPs). A fast and cost-effective way to identify bioactive metabolites in plant extracts prior to isolation has been developed based on the nuclear magnetic resonance (NMR) heterocovariance approach (NMR-HetCA). In order to evaluate in depth the application of this chemometrics based drug discovery methodology, simple mixtures of 10 standard NPs simulating a fast centrifugal partition chromatography (FCPC) fractionation (artificial fractions, ArtFrcts), as well as a more complex mixture of 59 natural standard substances simulating a crude plant extract (artificial extract, ArtExtr) were prepared. FCPC was employed for the fractionation of the ArtExtr, while the inhibitory activity of all fractions against DPPH was evaluated and their chemical profile was recorded using NMR spectroscopy. Spectral information was processed in the MATLAB environment and statistical approaches, including HetCA and statistical total correlation spectroscopy (STOCSY), were applied to identify bioactive compounds. Total heterocovariance plots (pseudospectra) facilitated the detection of highly correlated metabolites and led to the direct identification of 52.6% of the active compounds. The success in identifying the ArtExtr bioactive substances increased to 63.2% when spectral alignment was implemented. HetCA incorporates chromatographic (fractionation), spectroscopic (NMR profiling) and bioactivity results along with advanced chemometrics and could be established as a method of choice for the rapid and effective identification of bioactive NPs in plant extracts prior to isolation.

Table of contents

Solvents and reagents.......... 5

Fast centrifugal partition chromatography (FCPC).......... 5

Evaluation of free radical scavenging activity by DPPH assay.......... 5

High performance thin layer chromatography (HPTLC).......... 5

Table of Figures

Figure S1. Graphical representation of experimental design.......... 4

Figure S2. ArtExtr and ArtFrcts ingredients structures. The ArtFrcts ingredient structures are Trolox, **28** (Cm), **41** (Fr), **16** (Hsp), **15** (Nr), **03** (Qtn), **10** (Cf), **11** (Rm), **02** (Qtr) and **26** (Rt), respectively, based on Table S1. 7

Figure S3. Antioxidant activity against DPPH of the ArtFrcts at (a) 50 μg/mL and (b) 25 μg/mL. Results are expressed as the mean ±SD of three independent experiments. 11

Figure S4. Total HetCA plot resulted from the covariance of the NMR data with the biological activity of ArtFrcts in a concentration of 25 μg/mL and the annotation of the peaks belonging to the standard compounds. 12

Figure S5. HPTLC chromatograms of FCPC fractions. (a) NP at 254 nm; (b) NP in visible light after spraying with sulfuric vanillin reagent; (c) RP at 254 nm and (d) RP under white light after derivatization with vanillin sulfuric acid reagent.......... 13

Figure S6. Structures of the seven substances that were not included in the study of the ArtExtr. (a) **01**; (b) **09**; (c) **12**; (d) **13**; (e) **20**; (f) **31** and (g) **40**. 13

Figure S7. Example of misalignment of the peaks of compound **17** (nicotinic acid) in the fractions Fr30-42 (9.35-8.25 ppm).......... 15

Figure S8. Regions (a: 9.20-8.00, b: 7.90-7.30, c: 7.20-6.40, d: 6.35-5.90, e: 5.20-4.20, f: 4.10-3.50 and g: 3.20-2.00 ppm) of the Total HetCA pseudo-spectrum resulted from the covariance of the aligned ArtExtr fractions NMR data with their corresponding biological activity and the annotation of the peaks belonging to the compounds predicted as active. Numbering is according to Table S2. The left Y axis of each HetCA plot denotes the covariance, the right Y axis represents the correlation coefficient and the X axis indicates the ^{1}H-chemical shift (ppm).......... 17

Figure S9. Example of STOCSY pseudo-spectrum from signal at 6.62 ppm in ArtExtr fractions Fr20-60 (a: 10.00-0.50 and b: 8.00-6.50 ppm). The dark red peaks with correlation over 0.94 at δ_H 7.98 (dd), 7.55 (m), 6.72 (s) and 6.62 (s) correspond to **56** (baicalein), (c) Region (8.00-6.50 ppm) of the ^{1}H-NMR spectrum of standard baicalein. 18

Figure S10. Graphical representation of the results on the ArtExtr study. The gray text areas represent the concluded issues that emerged during the study. 19

Figure S11. Region of the Total HetCA plot (6.69-6.60 ppm), where one of the peaks of compound **38** (catechol) is displayed. 22

Figure S12. Concentration variance of compounds **04**, **16**, **38** and **56** (kaempferol, hesperetin, catechol and baicalein, respectively) in fractions Fr21-37 and % DPPH scavenging activity of respective fractions. 22

Figure S13. Partial HetCA plots (8.20-6.20 ppm) resulted from the covariance of biological activity against DPPH with corresponding NMR data of fractions (a) Fr25-29, (b) Fr26-30, (c) Fr27-31, (d) Fr28-32 and (e) Fr29-33. The highlighted peaks correspond to **38** (catechol). The left Y axis of each HetCA plot denotes the covariance, the right Y axis represents the correlation coefficient and the X axis indicates the ^{1}H-chemical shift (ppm). 23

Figure S14. a) Example of STOCSY pseudo-spectrum from signal at 6.38 (compound **02**, quercitrin) ppm in ArtExtr fractions Fr20-60 (10.0-0.8 ppm) and b) zoomed area (7.5-3.8 ppm). The dark red peaks with correlation over 0.94, correspond to compounds **02**, **05** and **59** (quercitrin, phlorizin and colchicine, respectively). 24

Figure S15. Concentration variance of compounds **02**, **05** and **59** (quercitrin, phlorizin and colchicine, respectively) in fractions Fr50-59... 24

Figure S16. Concentration variance of compounds **32** and **56** (caffeine and baicalein, respectively) in fractions Fr22-44. 25

Figure S17. Concentration variance of compounds **30** and **39** (ellagic acid and 3,5-dihydroxybenzoic acid, respectively) in fractions Fr48-55. 25

Figure S18. Concentration variance of compound **17** (nicotinic acid) and % DPPH scavenging activity of respective fractions. 26

Table of Tables

Table S1. Composition of the artificial fractions based on the mole fractions (%) of their constituents. 6

Table S2. Standard substances of the ArtExtr (name, code, molecular weight) and their scavenging activity against DPPH free radicals. 8

Table S3. Selected biphasic solvent systems for the FCPC stepwise elution-extrusion fractionation of the artificial extract (ArtExtr). 10

Table S4. Results of the *in vitro* antioxidant evaluation of the ArtFrcts standard compounds and the Total HetCA prediction at 25 μg/mL 11

Table S5. Results of Total HetCA in the ArtExtr fractions prior to spectra alignment. 13

Table S6. Results of Total HetCA in the ArtExtr fractions after spectra alignment. 15

Table S7. Approximate percentage content of each compound predicted as having an active contribution to the activity* in the respective fractions, based on the integration of their characteristic peaks 20

Table S8. Results of HetCA in series of five consecutive ArtExtr fractions (Fr20-70) after spectra alignment 26

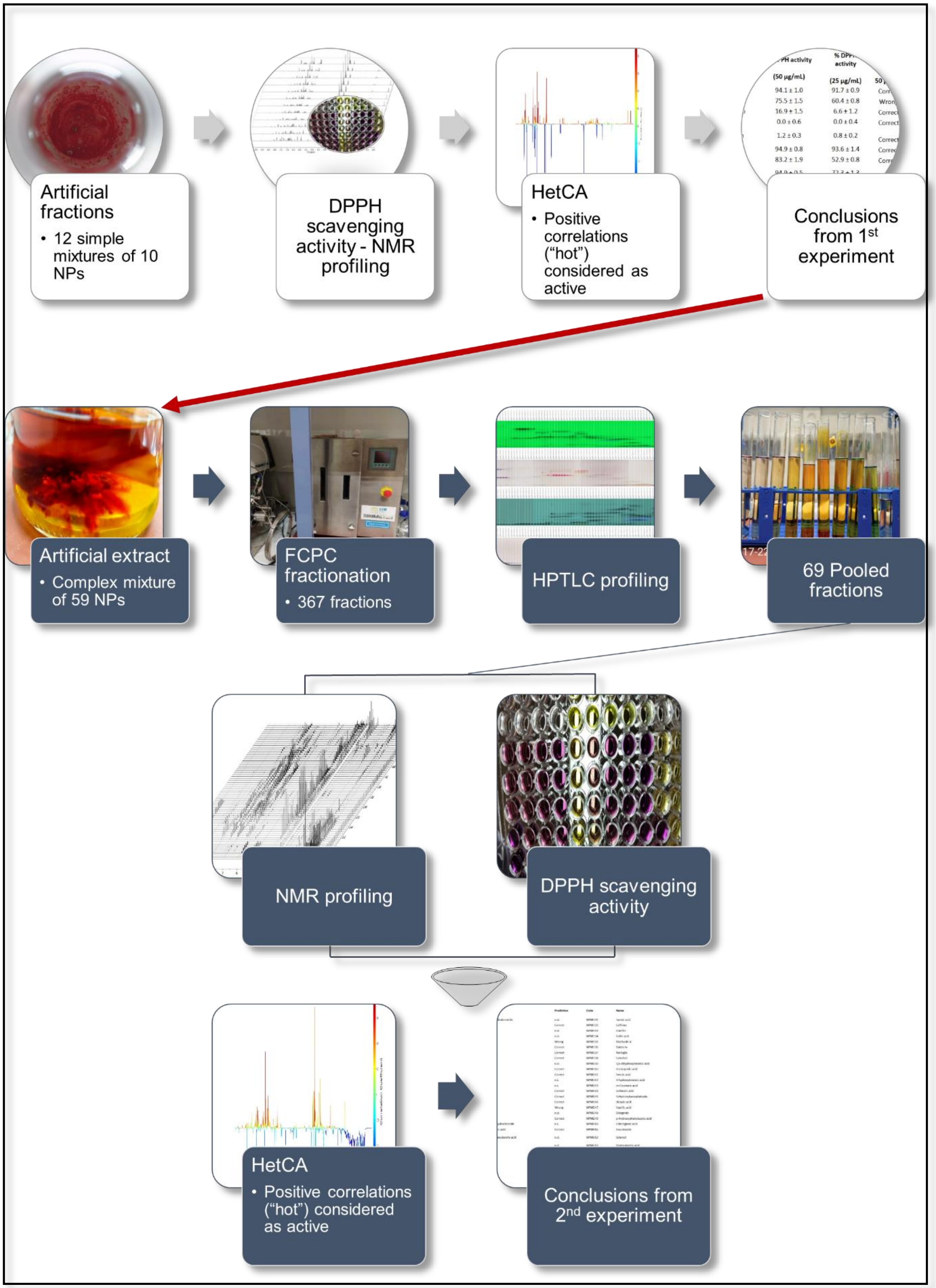

Figure S1. Graphical representation of experimental design.

Solvents and reagents

18 beta-glycyrrhetinic acid, 2,4-dimethoxyphenylacetic acid, 3,5-dihydroxybenzoic acid, 4-hydroxybenzaldehyde, 4-hydroxybenzoic acid, 6,7-dihydroxycoumarin (esculetin), arbutin, aristolochic acid, baicalein, biochanin A, caffeic acid, caffeine, catechol, chlorogenic acid, colchicine, *p*-coumaric acid, curcumin, daidzein, diosgenin, ellagic acid (dihydrate), ephedrine, ferulic acid, galantamine hydrobromide, gallic acid, harmine, hesperetin, homovanillic acid, *p*-hydroxyphenylacetic acid, kaempferol, naringenin, naringin, nicotinic acid, oleanolic acid, oleuropein, oxytetracycline hydrochloride, palmitic acid, protocatechic acid, quercetin, D-(−)-quinic acid, reserpine, resveratrol, rosmarinic acid, sclareol, shikonin, sinapic acid, sucrose, tannic acid, thymol, umbelliferone, vanillic acid and vanillin were purchased from Sigma-Aldrich (Sigma Aldrich, Steinheim, Germany). Aucubin, *m*-coumaric acid, isoferulic acid and loganin were purchased from Supelco (Supelco, Inc, Sigma Aldrich, Steinheim, Germany). (−)-Scopolamine methyl bromide, phlorizin quercitrin and rutin were purchased from PhytoLab (PhytoLab GmbH & Co. KG, Vestenbergsgreuth, Germany) and trolox from Acros Organics (Acros Organics B.V.B.A., Thermo Fisher Scientific, USA).

Fast centrifugal partition chromatography (FCPC)

The fractionation of the ArtExtr was performed by FCPC (FCPC KROMATON, France) with a 1000 mL column and adjustable rotation of 650-1700 rpm, equipped with a Gilson PLC 2250 pump and fraction collector compact system (Gilson Incorporated, Middleton, USA). The ArtExtr was fractionated using a step-gradient elution-extrusion normal phase method consisting of n-Hept, EtOAc, n-BuOH, MeOH and H_2O in ascending mode, starting with the mobile phase of S1 and gradually increasing the polarity by consecutively applying the mobile phases of S2-S8 (Table S3). More specifically, the 1000 mL column was initially filled with the stationary phase (i.e. the lower phase) of S1 (Table S3) at 650 rpm, with a flow rate of 50 mL/min. After increasing the rotation to 1200 rpm, the mobile phase of S1 (i.e. the upper phase) was pumped through the column with a flow rate of 10 mL/min. The equilibration was reached after 65 mL and the sample (1.2 g dissolved in 50 mL MeOH) was injected, while the fraction collector was set to 20 mL/fraction. The volume of each of the mobile phases used was 800 mL, so 40 fractions/mobile phase were collected. Elution extrusion was performed with the lower phase of S8 with a flow rate of 30 mL/min at 1700 rpm and 36 fractions were collected. The extrusion was completed by applying 2 L of the lower phase of S8 which was collected in a flask and not included in the results. The total number of fractions collected was 367, which were then pooled to 69 fractions based on their TLC profiles, aiming to the optimal variance of the included compounds. The resulting 69 pooled fractions were diluted in methanol at a concentration level of 4 mg/mL, filtered with polytetrafluoroethylene (PTFE) filters (13 mm × 22 μm, RephiLe Bioscience Ltd, USA) and solvents' evaporation was achieved under reduced pressure using an RVC 2-33 CDplus evaporator system equipped with an Alpha 2-4 LSCbasic freeze dryer (Martin Christ, Germany). The filtered samples were forwarded for NMR-HetCA, HPTLC profiling and *in vitro* antioxidant assay evaluation.

Evaluation of free radical scavenging activity by DPPH assay

The screening of the antioxidant activity of the standard substances and, subsequently, the FCPC fractions was estimated by the DPPH assay as previously described by Lee *et al.*[47]. Samples were diluted at a stock concentration level of 10 mg/mL in DMSO and 10 μL of each sample were mixed with 190 μL of DPPH solution (≈ 0.317 mM, 12.5 mg DPPH/100 mL EtOH) in a 96-well plate and then subsequently incubated, at room temperature, for 30 min in darkness. Finally, the absorbance was measured at 517 nm, using the Tecan Infinite M1000 PRO reader (Tecan Austria GmbH), while the system was operating under the Tecan i-control v.1.11. All evaluations were performed in triplicates, while gallic acid was used as positive control (IC_{50}= 30.2 μM). The % inhibition of the DPPH radical for each dilution was calculated using the following formula:

$$\%Inhibition = \{[1-(A_S-A_B)]/A_C\}\times 100$$

where A_S is the absorbance of the sample, A_C the absorbance of the control and A_B the absorbance of the sample without the DPPH radical. Inhibition was expressed as mean ± SD (N=3). The *in vitro* DPPH inhibition assay for the ArtFrcts, as well as for the standards comprising them, was performed at 50 μg/mL and 25 μg/mL. The standard substances comprising the ArtExtr were evaluated *in vitro* at 100 μg/mL and IC_{50} values were calculated for the most active ones. Regarding the ArtExtr FCPC fractions, the evaluation took place at a final concentration of 75 μg/mL in the well.

High performance thin layer chromatography (HPTLC)

For the chemical profiling of the FCPC fractions of the artificial extract, the filtered samples were re-diluted in methanol at a concentration level of 3 mg/mL. Subsequently, 7 μL of each sample were applied on HPTLC normal phase aluminum plates, (20 × 10 cm) precoated with silica gel 60 F_{254} (150–200 mm) and HPTLC reversed aluminum plates, (20 × 10 cm) precoated with silica gel 60 RP-18 F_{254S} (150–200 mm) (Merck, Darmstadt, Germany) as 7 mm bands, using an automatic TLC Sampler 4 (ATS-4, CAMAG, Muttenz, Switzerland). The chromatographic separation was performed in the Automatic Developing Chamber 2 (ADC 2) with a solvent system consisting of Tol, EtOAc and Fa (60/40/1 v/v/v) for the normal phase and H_2O, MeCN and Fa (70/30/1 v/v/v) for the reversed phase, up to a migration distance of 75 mm (from the lower plate edge). The same conditions were used for the development of all the plates. The plates were then documented under 254 nm, 366

nm and at white light after derivatization with the vanillin–sulphuric acid reagent with CAMAG Visualizer 2. The system was operating under the VisionCats 3.0 software (CAMAG).

Table S1. Composition of the artificial fractions based on the mole fractions (%) of their constituents.

Compound/Fraction	Fr1	Fr2	Fr3	Fr4	Fr5	Fr6	Fr7	Fr8	Fr9	Fr10	Fr11	Fr12
**Trolox (Tr)**	100.0	61.7	23.1	0.0	0.0	0.0	0.0	0.0	0.0	0.0	0.0	0.0
**p-coumaric acid (Cm)**	0.0	26.9	47.1	49.0	38.6	14.8	0.0	0.0	0.0	0.0	0.0	0.0
**Ferulic acid (Fr)**	0.0	11.4	29.8	31.1	32.6	37.4	26.5	13.9	0.0	0.0	0.0	0.0
**Hesperetin (Hsp)**	0.0	0.0	0.0	20.0	21.0	24.1	8.6	0.0	0.0	0.0	0.0	0.0
**Naringenin (Nr)**	0.0	0.0	0.0	0.0	7.8	8.9	19.0	9.9	0.0	0.0	0.0	0.0
**Quercetin (Qtn)**	0.0	0.0	0.0	0.0	0.0	8.0	17.1	17.9	9.6	0.0	0.0	0.0
**Caffeic acid (Cf)**	0.0	0.0	0.0	0.0	0.0	0.0	14.4	29.8	48.4	50.9	51.8	41.0
**Rosmarinic acid (Rm)**	0.0	0.0	0.0	0.0	0.0	6.7	14.4	22.5	24.2	25.5	25.9	20.6
**Quercitrin (Qtr)**	0.0	0.0	0.0	0.0	0.0	0.0	0.0	6.0	13.0	13.6	6.9	8.2
**Rutin (Rt)**	0.0	0.0	0.0	0.0	0.0	0.0	0.0	0.0	4.8	10.0	15.4	30.2

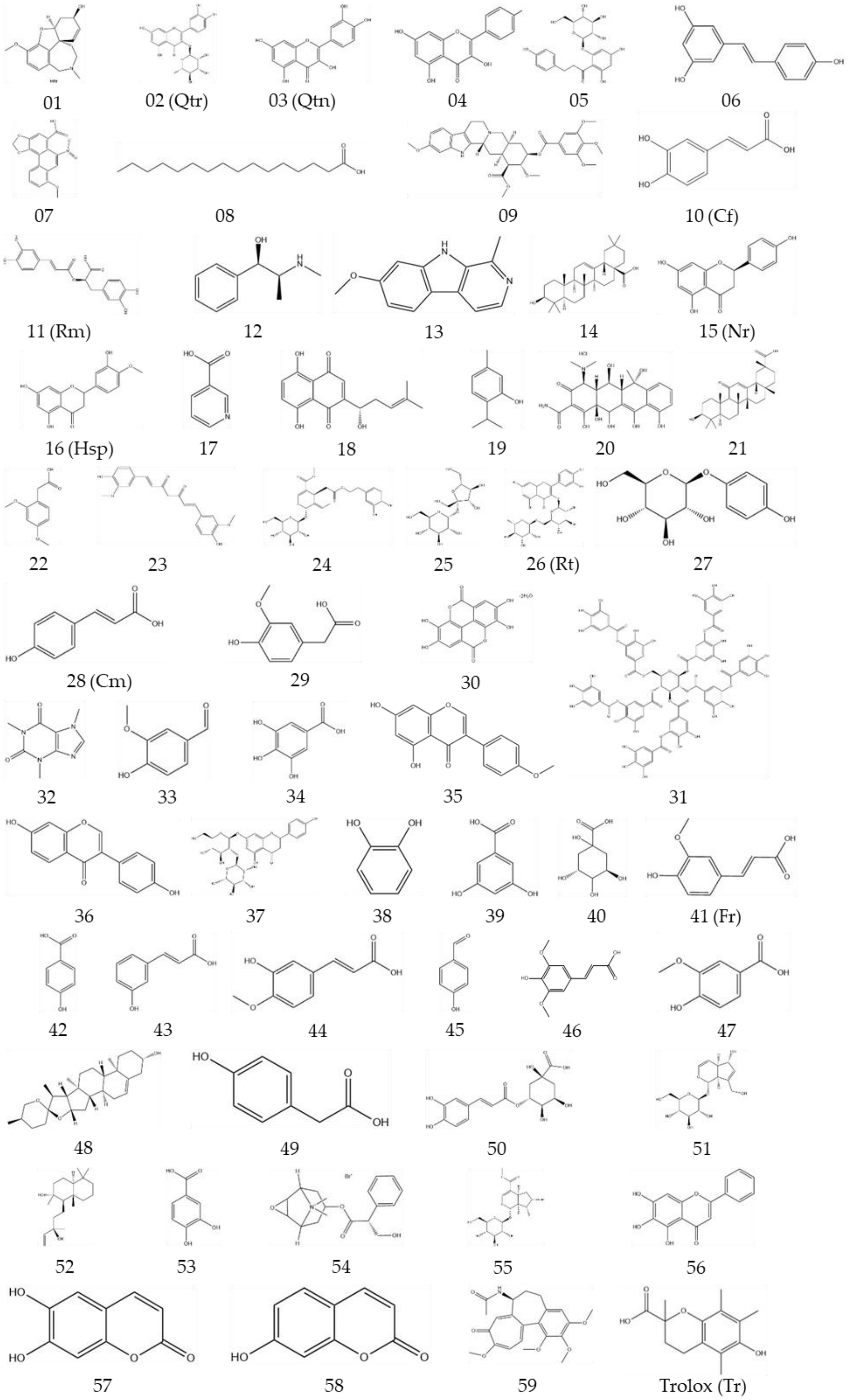

Figure S2. ArtExtr and ArtFrcts ingredients structures. The ArtFrcts ingredient structures are Trolox, **28** (Cm), **41** (Fr), **16** (Hsp), **15** (Nr), **03** (Qtn), **10** (Cf), **11** (Rm), **02** (Qtr) and **26** (Rt), respectively, based on Table S1.

Table S2. Standard substances of the ArtExtr (name, code, molecular weight) and their scavenging activity against DPPH free radicals.

Code	Name	Chemical category	DPPH activity (100 μg/mL)	IC_{50}^{*} (μM)	Molecular Weight (g/mol)	mmoles	Molecular fraction (%) in AE
01	Galanthamine hydrobromide	Alkaloid	1.2 ± 0.4		368.30	0.14	1.13
02	Quercitrin	Flavonoid	91.4 ± 0.2	50.5	448.40	0.11	0.89
03	Quercetin	Flavonoid	96.6 ± 0.1	30.3	302.23	0.17	1.37
04	Kaempferol	Flavonoid	96.7 ± 0.0	68.1	286.24	0.17	1.37
05	Phlorizin	Chalcone	0.0 ± 0.6		436.40	0.11	0.89
06	Resveratrol	Stilbenoid	74.5 ± 0.7	225.4	228.24	0.22	1.78
07	Aristolochic acid	Monocarboxylic acid	0.0 ± 1.1		341.27	0.15	1.21
08	Palmitic acid	Fatty acid	0.0 ± 1.2		256.42	0.19	1.53
09	Reserpine	Alkaloid	0.0 ± 1.0		608.70	0.08	0.65
10	Caffeic acid	Phenolic acid	95.8 ± 0.1	44.7	180.16	0.28	2.26
11	Rosmarinic acid	Phenolic acid	95.8 ± 0.0	37.7	360.30	0.14	1.13
12	Ephedrine	Alkaloid	0.0 ± 0.4		165.23	0.30	2.42
13	Harmine	Alkaloid	0.0 ± 0.8		212.25	0.24	1.94
14	Oleanolic acid	Terpenoid	0.0 ± 0.4		456.70	0.11	0.89
15	Naringenin	Flavonoid	0.0 ± 0.4		272.25	0.18	1.45
16	Hesperetin	Flavonoid	40.2 ± 0.4		302.28	0.17	1.37
17	Nicotinic acid	Pyridinecarboxylic acid	0.4 ± 0.5		123.11	0.41	3.31
18	Shikonin	Naphthoquinone	41.0 ± 3.1		288.29	0.17	1.37
19	Thymol	Phenol	18.6 ± 0.6		150.22	0.33	2.66
20	Oxytetracycline hydrochloride	Tetracycline (Alkaloid)	8.0 ± 0.5		496.90	0.10	0.81
21	18-β-glycyrrhetinic acid	Terpenoid	0.0 ± 1.6		470.70	0.11	0.89

22	2,4-dimethoxy-phenylacetic acid	Phenolic acid	0.0 ± 0.4		196.20	0.25	2.02
23	Curcumin	Polyphenol	95.6 ± 0.2	80.8	368.40	0.14	1.13
24	Oleuropein	Iridoid	96.2 ± 0.0	61.1	540.50	0.09	0.73
25	Sucrose	Sugar	0.0 ± 0.7		342.30	0.15	1.21
26	Rutin	Flavonoid	91.6 ± 0.0	46.8	610.50	0.08	0.65
27	Arbutin	Phenol	41.1 ± 0.5		272.25	0.18	1.45
28	*p*-Coumaric acid	Phenolic acid	1.4 ± 0.5		164.16	0.30	2.42
29	Homovanillic acid	Phenolic acid	66.5 ± 0.3	261.3	182.17	0.27	2.18
30	Ellagic acid (dihydrate)	Polyphenol	95.7 ± 0.0	20.8	338.22	0.15	1.21
31	Tannic acid	Polyphenol	96.4 ± 0.1	3.1	1701.20	0.03	0.24
32	Caffeine	Xanthine	0.0 ± 0.8		194.19	0.26	2.10
33	Vanillin	Benzaldehyde	2.4 ± 0.5		152.15	0.33	2.66
34	Gallic acid	Phenolic acid	95.7 ± 0.0	30.2	170.12	0.29	2.34
35	Biochanin A	Flavonoid	0.0 ± 0.4		284.26	0.18	1.45
36	Daidzein	Flavonoid	0.0 ± 0.3		254.24	0.20	1.61
37	Naringin	Flavonoid	0.0 ± 1.3		580.50	0.09	0.73
38	Catechol	Phenol	95.9 ± 0.1	48.2	110.11	0.45	3.63
39	3,5-dihydroxybenzoic acid	Phenolic acid	1.0 ± 0.3		154.12	0.32	2.58
40	D-(−)-quinic acid	Cyclitol	0.0 ± 0.9		192.17	0.26	2.10
41	Ferulic acid	Phenolic acid	90.2 ± 0.3	95.2	194.18	0.26	2.10
42	4-hydroxybenzoic acid	Phenolic acid	0.0 ± 0.6		138.12	0.36	2.91
43	*m*-Coumaric acid	Phenolic acid	2.2 ± 2.3		164.16	0.30	2.42
44	Isoferulic acid	Phenolic acid	22.4 ± 0.1		194.18	0.26	2.10
45	4-Hydroxybenzaldehyde	Benzaldehyde	0.0 ± 1.2		122.12	0.41	3.31

46	Sinapic acid	Phenolic acid	93.5 ± 0.1	82.2	224.21	0.22	1.78
47	Vanillic acid	Phenolic acid	6.1 ± 0.1		168.15	0.30	2.42
48	Diosgenin	Terpenoid	0.0 ± 1.0		414.60	0.12	0.97
49	*p*-Hydroxy-phenylacetic acid	Phenolic acid	0.0 ± 0.7		152.15	0.33	2.66
50	Chlorogenic acid	Cyclitol	91.7 ± 0.5	87.9	354.31	0.14	1.13
51	Aucuboside	Iridoid	0.0 ± 0.9		346.33	0.14	1.13
52	Sclareol	Terpenoid	0.0 ± 0.0		308.50	0.16	1.29
53	Protocatechic acid	Phenolic acid	92.7 ± 0.3	110.6	154.12	0.32	2.58
54	(−)-Scopolamine methyl bromide	Alkaloid	0.0 ± 0.6		398.30	0.13	1.05
55	Loganin	Iridoid	0.0 ± 0.5		390.40	0.13	1.05
56	Baicalein	Flavonoid	95.4 ± 0.0	26.3	270.24	0.19	1.53
57	6,7-Dihy-droxycoumarin (Esculetin)	Coumarin	95.2 ± 0.0	27.8	178.14	0.28	2.26
58	Umbelliferone	Coumarin	0.0 ± 0.4		162.14	0.31	2.50
59	Colchicine	Alkaloid	0.0 ± 0.3		399.40	0.13	1.05

*IC_{50}: half-maximal inhibitory concentration. Results are expressed as the mean ±SD of three independent experiments.

Table S3. Selected biphasic solvent systems for the FCPC stepwise elution-extrusion fractionation of the artificial extract (ArtExtr).

	n-Hept	EtOAc	n-BuOH	MeOH	H_2O
S1	9	1	0	5	5
S2	8	2	0	5	5
S3	7	3	0	5	5
S4	6	4	0	5	5
S5	5	5	0	5	5
S6	2	8	0	5	5
S7	2	8	1	4	5
S8	2	8	2	3	5

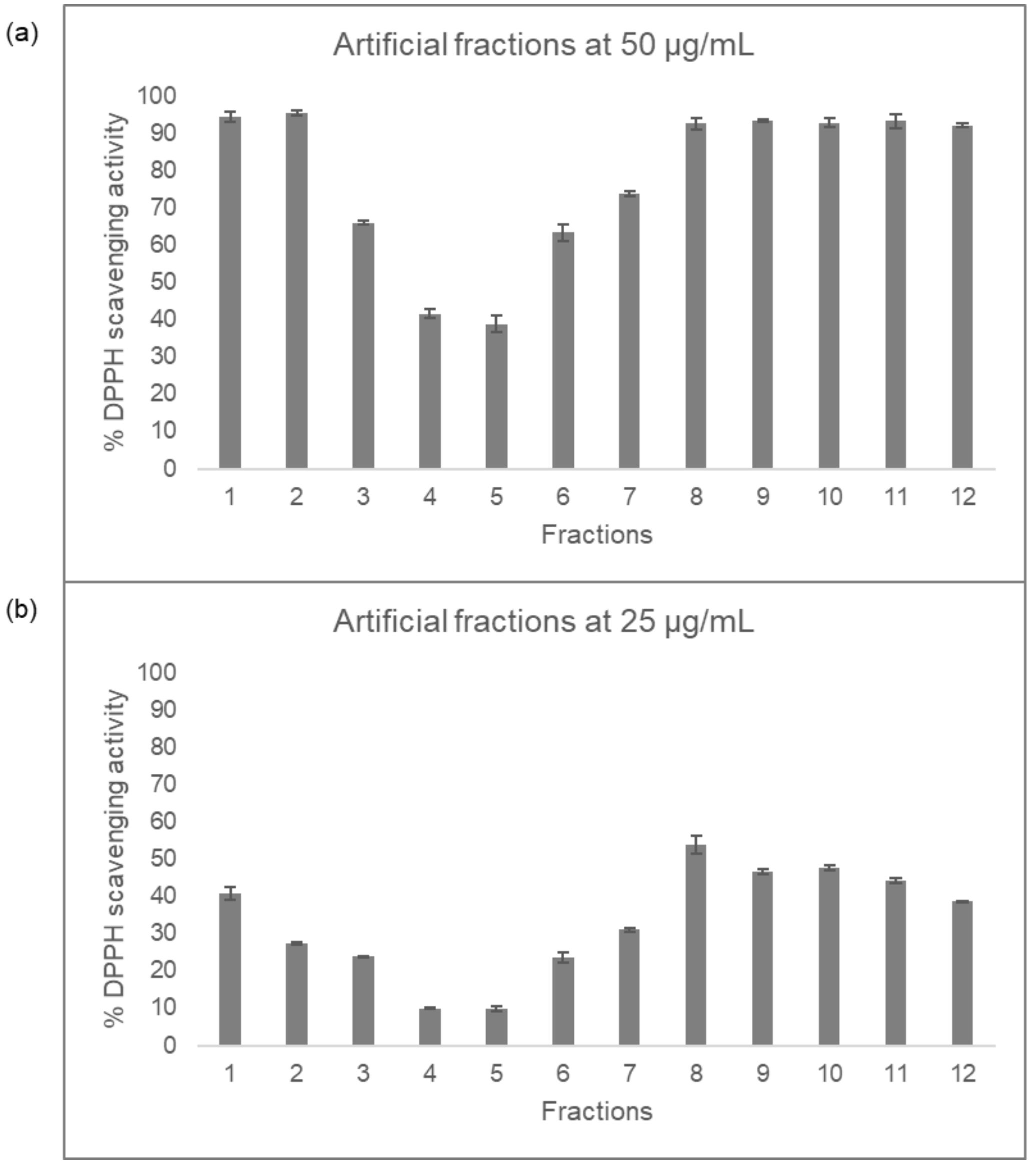

Figure S3. Antioxidant activity against DPPH of the ArtFrcts at (a) 50 µg/mL and (b) 25 µg/mL. Results are expressed as the mean ±SD of three independent experiments.

Table S4. Results of the *in vitro* antioxidant evaluation of the ArtFrcts standard compounds and the Total HetCA prediction at 25 µg/mL.

Standard	Code	% DPPH activity (50 µg/mL)	% DPPH activity (25 µg/mL)	HetCA prediction – 25 µg/mL
Caffeic acid	Cf	94.1 ± 1.0	91.7 ± 0.9	Correct
Ferulic acid	Fr	75.5 ± 1.5	60.4 ± 0.8	False negative
Hesperetin	Hsp	16.9 ± 1.5	6.6 ± 1.2	Correct
Naringenin	Nr	0.0 ± 0.6	0.0 ± 0.4	Correct

p-coumaric acid	Cm	1.2 ± 0.3	0.8 ± 0.2	Correct
Quercetin	Qtn	94.9 ± 0.8	93.6 ± 1.4	Correct
Quercitrin	Qtr	83.2 ± 1.9	52.9 ± 0.8	Correct
Rosmarinic acid	Rm	94.9 ± 0.5	72.3 ± 1.3	Correct
Rutin	Rt	84.1 ± 1.8	45.4 ± 0.6	Correct
Trolox	Tr	92.0 ± 1.5	26.4 ± 0.7	Correct

Results are expressed as the mean ±SD of three independent experiments.

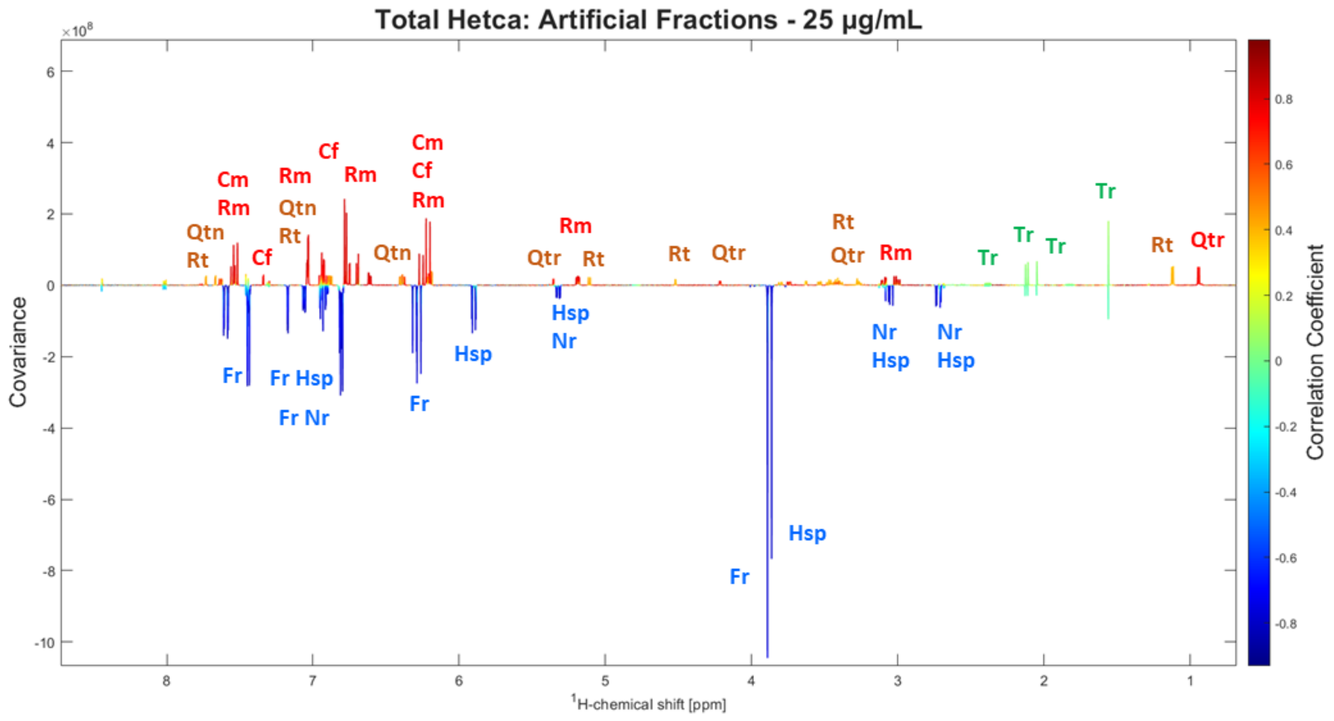

Figure S4. Total HetCA plot resulted from the covariance of the NMR data with the biological activity of ArtFrcts in a concentration of 25 μg/mL and the annotation of the peaks belonging to the standard compounds.

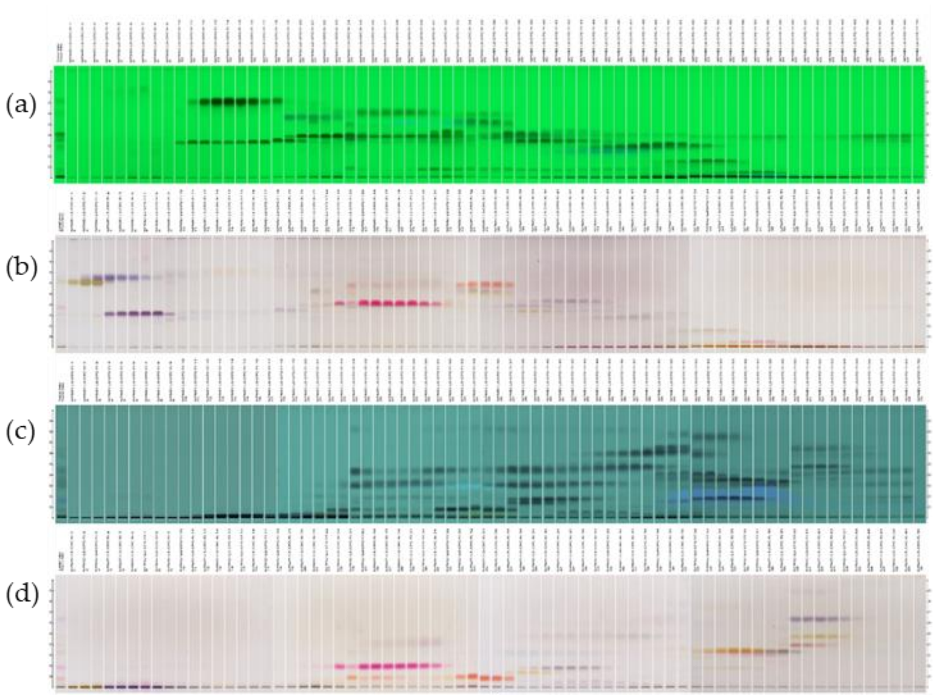

Figure S5. HPTLC chromatograms of FCPC fractions. (a) NP at 254 nm; (b) NP in visible light after spraying with sulfuric vanillin reagent; (c) RP at 254 nm and (d) RP under white light after derivatization with vanillin sulfuric acid reagent.

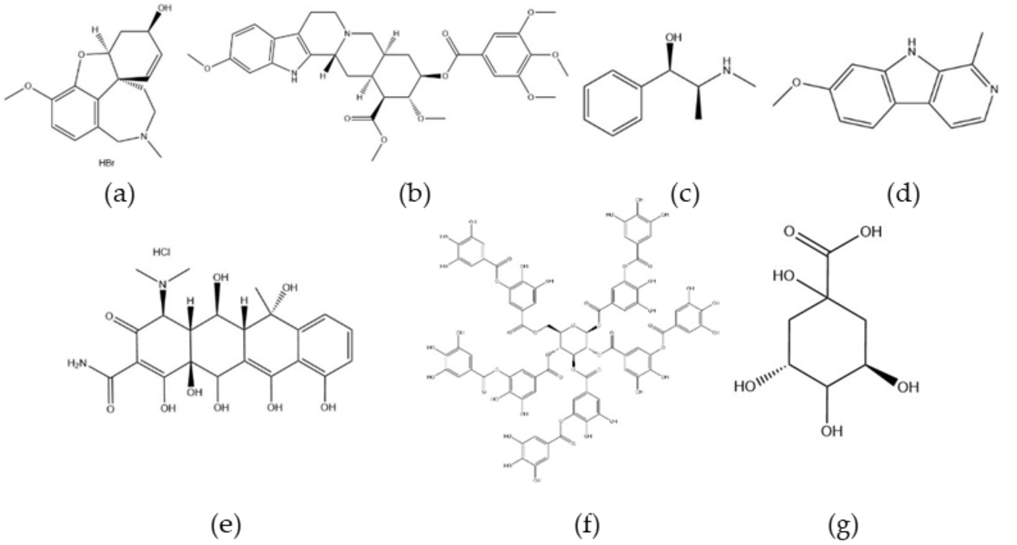

Figure S6. Structures of the seven substances that were not included in the study of the ArtExtr. (a) **01**; (b) **09**; (c) **12**; (d) **13**; (e) **20**; (f) **31** and (g) **40**.

Table S5. Results of Total HetCA in the ArtExtr fractions prior to spectra alignment.

Code	Name	Prediction	Code	Name	Prediction
01	Galanthamine hydrobromide	n.i.	31	Tannic acid	n.i.

02	Quercitrin	ND	32	Caffeine	False positive
03	Quercetin	ND	33	Vanillin	Correct
04	Kaempferol	ND	34	Gallic acid	Correct
05	Phlorizin	False positive	35	Biochanin A	Correct
06	Resveratrol	Correct	36	Daidzein	ND
07	Aristolochic acid	ND	37	Naringin	ND
08	Palmitic acid	Correct	38	Catechol	Correct
09	Reserpine	n.i.	39	3,5-dihydroxybenzoic acid	False positive
10	Caffeic acid	Correct	40	D-(−)-quinic acid	n.i.
11	Rosmarinic acid	ND	41	Ferulic acid	ND
12	Ephedrine	n.i.	42	4-hydroxybenzoic acid	False positive
13	Harmine	n.i.	43	*m*-Coumaric acid	ND
14	Oleanolic acid	Correct	44	Isoferulic acid	ND
15	Naringenin	ND	45	4-Hydroxybenzaldehyde	False positive
16	Hesperetin	ND	46	Sinapic acid	ND
17	Nicotinic acid	False positive	47	Vanillic acid	ND
18	Shikonin	ND	48	Diosgenin	Correct
19	Thymol	Correct	49	*p*-Hydroxyphenylacetic acid	ND
20	Oxytetracycline hydrochloride	n.i.	50	Chlorogenic acid	ND
21	18-β-glycyrrhetinic acid	Correct	51	Aucuboside	ND
22	2,4-dimethoxyphenylacetic acid	False positive	52	Sclareol	Correct
23	Curcumin	ND	53	Protocatechic acid	Correct
24	Oleuropein	Correct	54	(−)-Scopolamine methyl bromide	Correct
25	Sucrose	Correct	55	Loganin	ND
26	Rutin	Correct	56	Baicalein	Correct
27	Arbutin	False positive	57	6,7-Dihydroxycoumarin (Esculetin)	Correct
28	*p*-Coumaric acid	ND	58	Umbelliferone	Correct

29	Homovanillic acid	ND	59	Colchicine	False positive
30	Ellagic acid (dihydrate)	Correct			

n.i.: Not included in the study, ND: Not detected

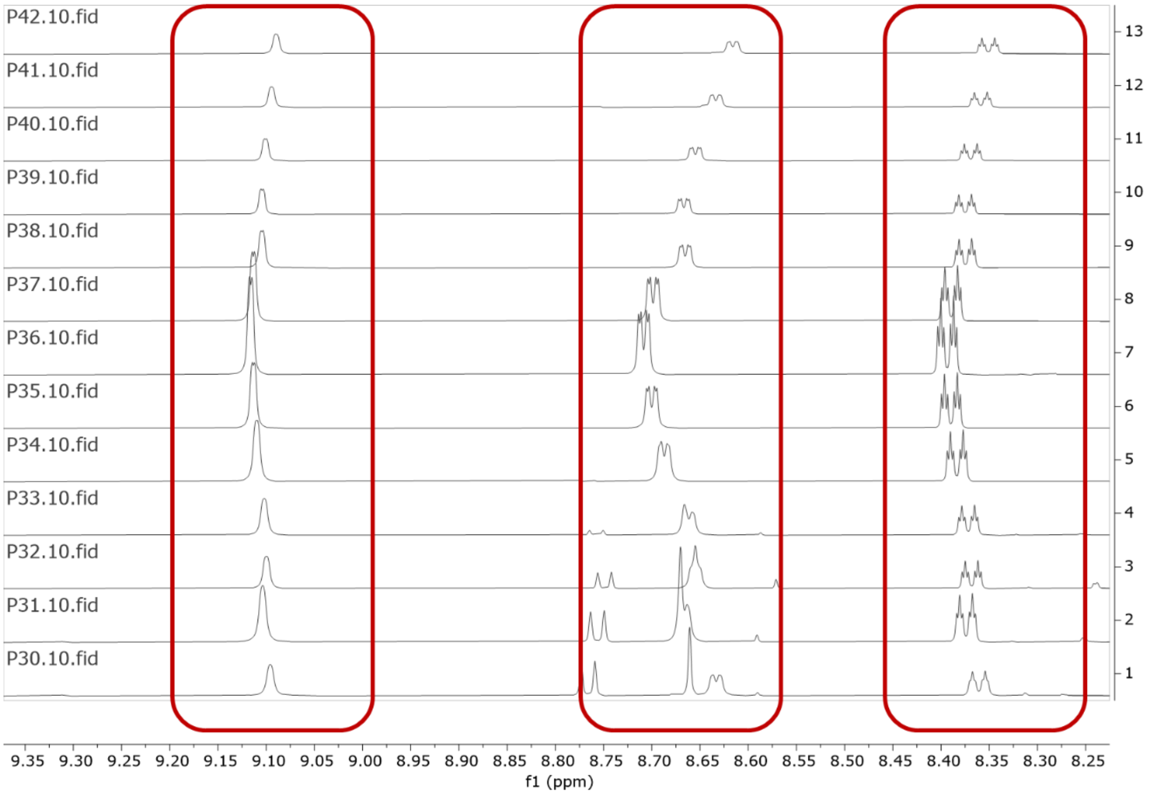

Figure S7. Example of misalignment of the peaks of compound **17** (nicotinic acid) in the fractions Fr30-42 (9.35-8.25 ppm).

Table S6. Results of Total HetCA in the ArtExtr fractions after spectra alignment.

Code	Name	Prediction	Code	Name	Prediction
01	Galanthamine hydrobromide	n.i.	31	Tannic acid	n.i.
02	Quercitrin	Correct	32	Caffeine	False positive
03	Quercetin	ND	33	Vanillin	Correct
04	Kaempferol	ND	34	Gallic acid	Correct
05	Phlorizin	False positive	35	Biochanin A	Correct
06	Resveratrol	Correct	36	Daidzein	ND

07	Aristolochic acid	Correct	37	Naringin	ND
08	Palmitic acid	Correct	38	Catechol	False negative
09	Reserpine	n.i.	39	3,5-dihydroxybenzoic acid	False positive
10	Caffeic acid	Correct	40	D-(−)-quinic acid	n.i.
11	Rosmarinic acid	Correct	41	Ferulic acid	ND
12	Ephedrine	n.i.	42	4-hydroxybenzoic acid	Correct
13	Harmine	n.i.	43	*m*-Coumaric acid	ND
14	Oleanolic acid	Correct	44	Isoferulic acid	ND
15	Naringenin	Correct	45	4-Hydroxybenzaldehyde	Correct
16	Hesperetin	Correct	46	Sinapic acid	Correct
17	Nicotinic acid	False positive	47	Vanillic acid	ND
18	Shikonin	ND	48	Diosgenin	Correct
19	Thymol	Correct	49	*p*-Hydroxyphenylacetic acid	ND
20	Oxytetracycline hydrochloride	n.i.	50	Chlorogenic acid	ND
21	18-β-glycyrrhetinic acid	Correct	51	Aucuboside	ND
22	2,4-dimethoxyphenylacetic acid	ND	52	Sclareol	Correct
23	Curcumin	ND	53	Protocatechic acid	Correct
24	Oleuropein	Correct	54	(−)-Scopolamine methyl bromide	ND
25	Sucrose	Correct	55	Loganin	ND
26	Rutin	Correct	56	Baicalein	Correct
27	Arbutin	Correct	57	6,7-Dihydroxycoumarin (Esculetin)	Correct
28	*p*-Coumaric acid	ND	58	Umbelliferone	ND
29	Homovanillic acid	ND	59	Colchicine	False positive
30	Ellagic acid (dihydrate)	Correct			

n.i.: Not included in the study, ND: Not detected

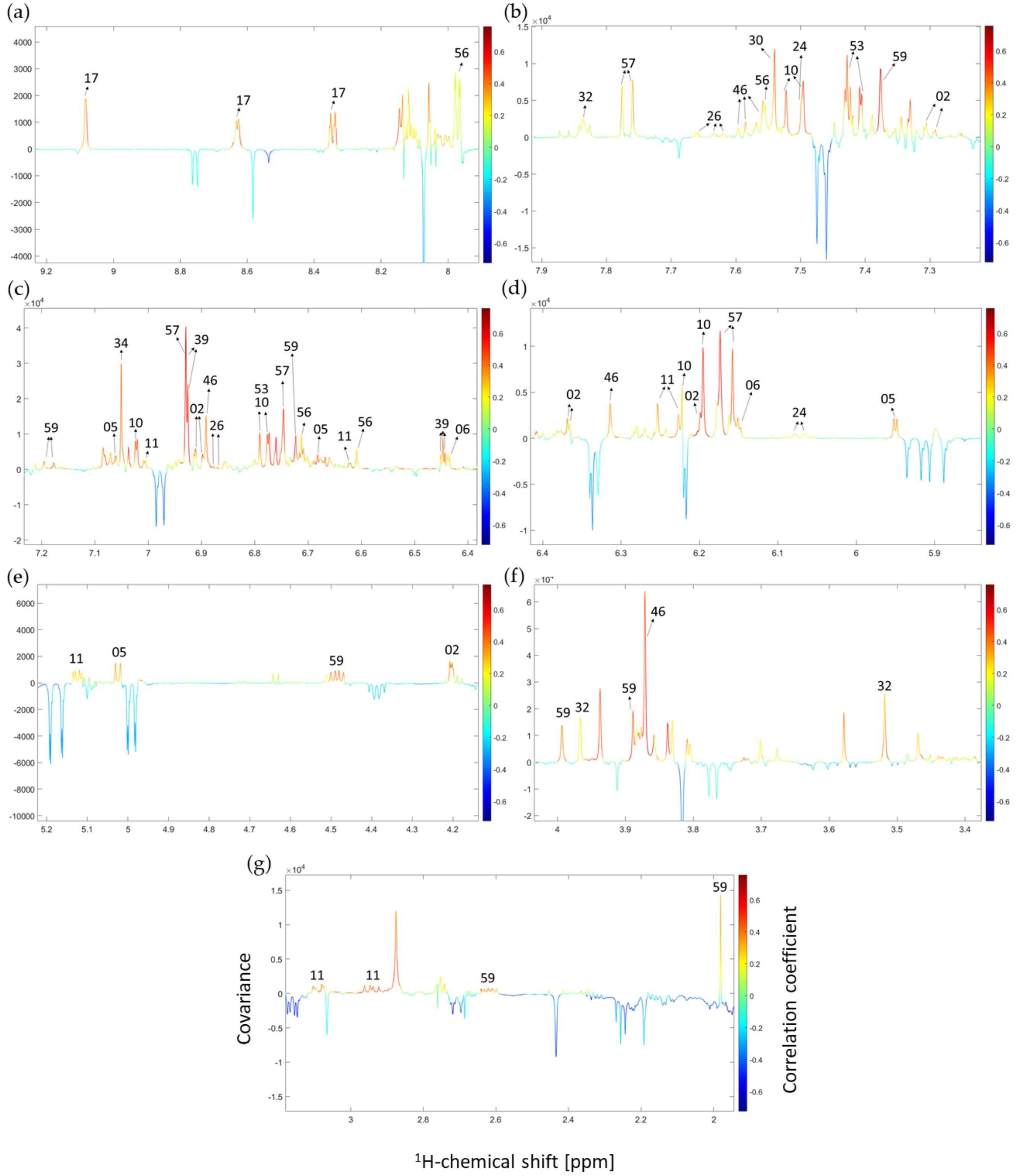

Figure S8. Regions (a: 9.20-8.00, b: 7.90-7.30, c: 7.20-6.40, d: 6.35-5.90, e: 5.20-4.20, f: 4.10-3.50 and g: 3.20-2.00 ppm) of the Total HetCA pseudo-spectrum resulted from the covariance of the aligned ArtExtr fractions NMR data with their corresponding biological activity and the annotation of the peaks belonging to the compounds predicted as active. Numbering is according to Table S2. The left Y axis of each HetCA plot denotes the covariance, the right Y axis represents the correlation coefficient and the X axis indicates the ^{1}H-chemical shift (ppm).

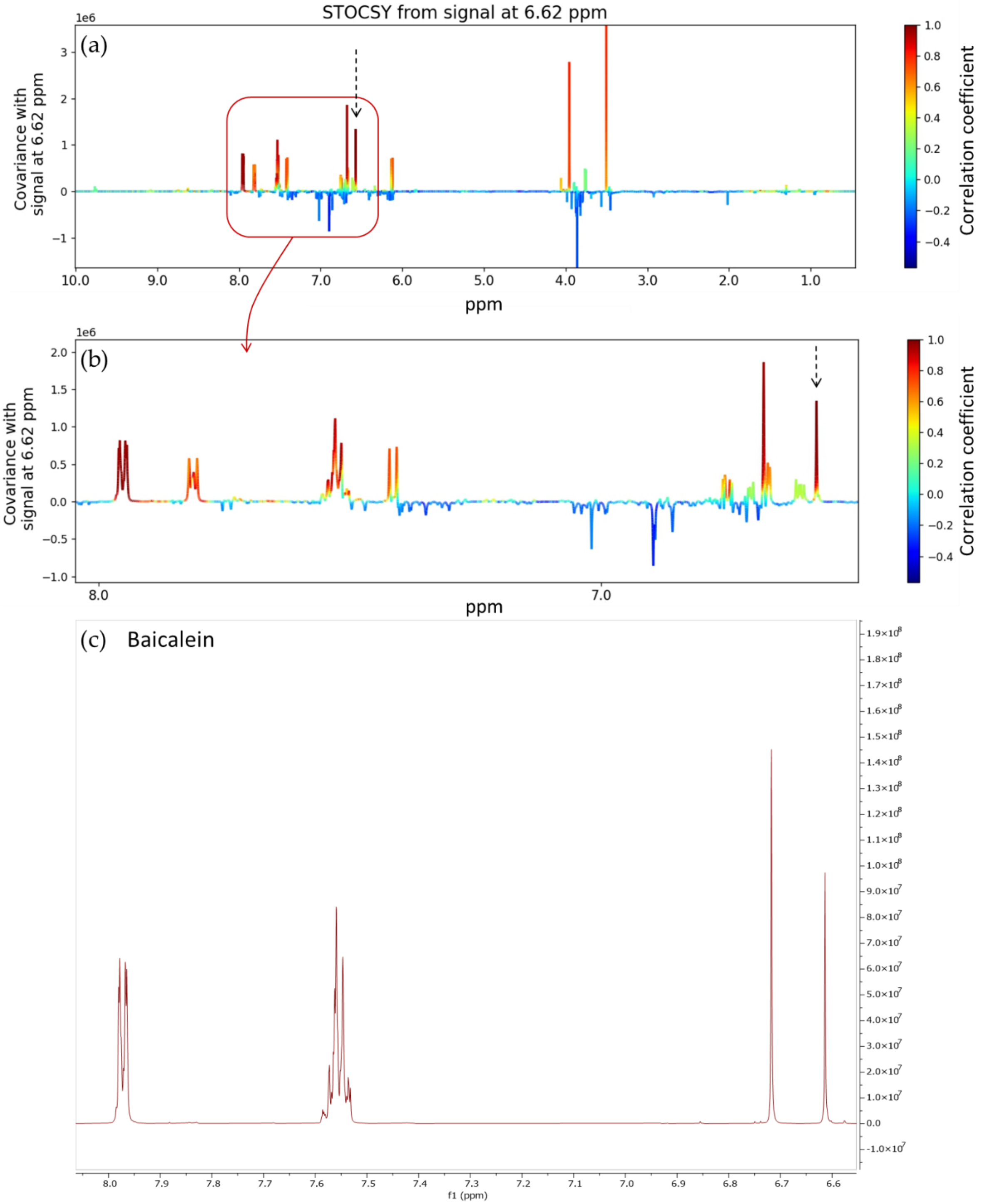

Figure S9. Example of STOCSY pseudo-spectrum from signal at 6.62 ppm in ArtExtr fractions Fr20-60 (a: 10.00-0.50 and b: 8.00-6.50 ppm). The dark red peaks with correlation over 0.94 at δ_H 7.98 (dd), 7.55 (m), 6.72 (s) and 6.62 (s) correspond to **56** (baicalein), (c) Region (8.00-6.50 ppm) of the ^{1}H-NMR spectrum of standard baicalein.

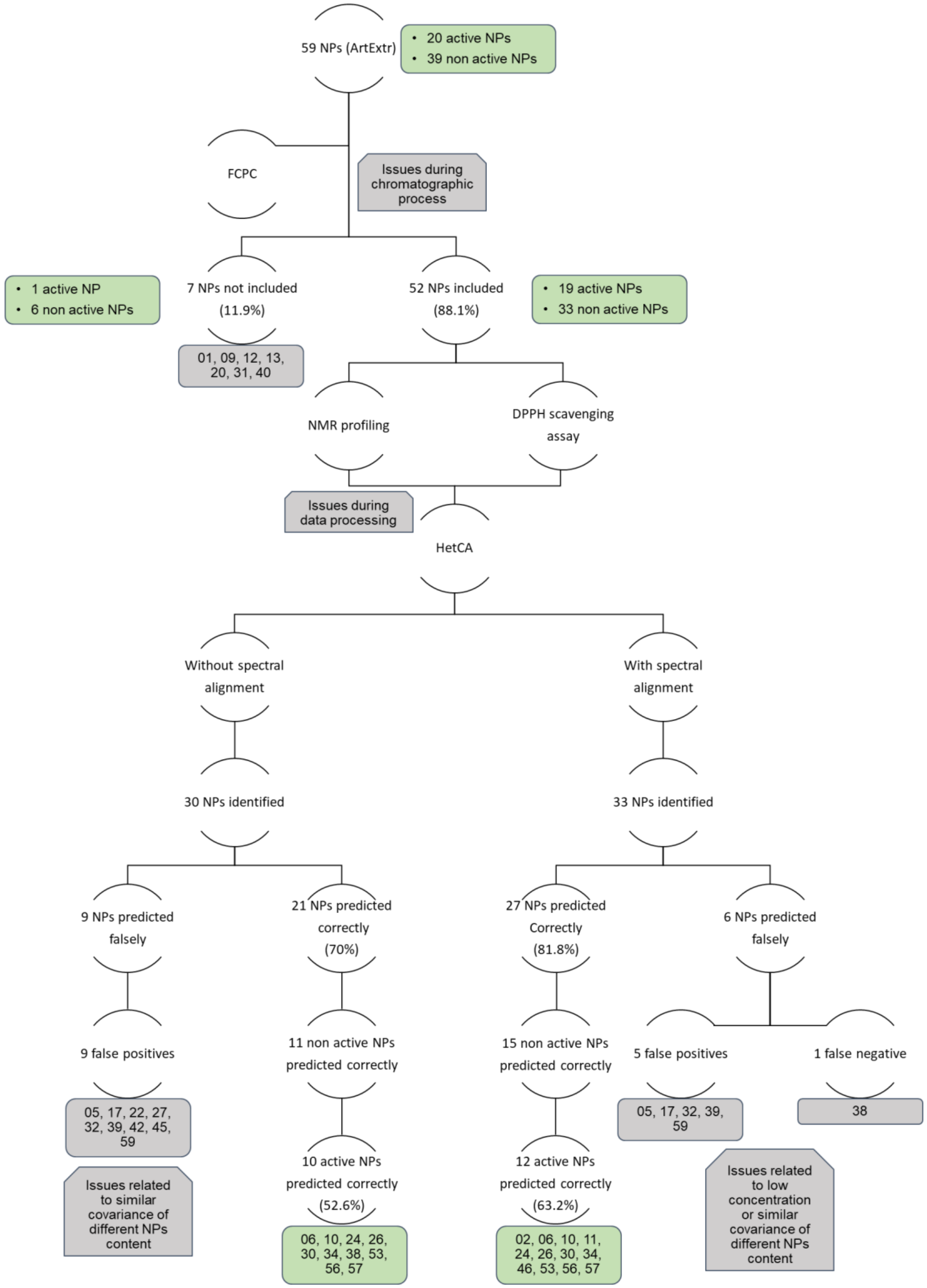

Figure S10. Graphical representation of the results on the ArtExtr study. The gray text areas represent the concluded issues that emerged during the study.

Table S7. Approximate percentage content of each compound predicted as having an active contribution to the activity* in the respective fractions, based on the integration of their characteristic peaks.

Compound	02	05	06	10	11	17	24	26	30	32	34	39	46	53	56	57	59
NMR integrated Peak (ppm)	5.35	5.03	6.45	7.53	7.49	9.10	6.10	7.67	7.54	3.97	7.06	6.47	6.33	7.44-7.42	7.99	7.77	4.50
Fr 21	0	0	0	0	0	0	0	0	0	0	0	0	0	0	0	0	0
Fr 22	0	0	0	0	0	0	0	0	0	0	0	0	0	0	43.8	0	0
Fr 23	0	0	0	0	0	0	0	0	0	0	0	0	0	0	32.2	0	0
Fr 24	0	0	0	0	0	0	0	0	0	0	0	0	0	0	12.6	0	0
Fr 25	0	0	0	0	0	0	0	0	0	0	0	0	0	0	7.8	0	0
Fr 26	0	0	0	0	0	0	0	0	0	1.5	0	0	0	0	4.5	0	0
Fr 27	0	0	0	0	0	0	0	0	0	1.7	0	0	0	0	2.2	0	0
Fr 28	0	0	0	0	0	0	0	0	0	1.5	0	0	0	0	1.2	0	0
Fr 29	0	0	0	0	0	0	0	0	0	3.7	0	0	0	0	1.5	0	0
Fr 30	0	0	0	0	0	2.7	0	0	0	25.1	0	0	0	0	2.0	0	0
Fr 31	0	0	0	0	0	3.6	0	0	0	40.6	0	0	0	0	19.8	0	0
Fr 32	0	0	0	0	0	2.4	0	0	0	33.3	0	0	0	0	45.9	0	0
Fr 33	0	0	0	0	0	2.9	0	0	0	23.2	0	0	0	0	32.7	0	0
Fr 34	0	0	0	0	0	5.6	0	0	0	14.5	0	0	0	0	8.7	0	0
Fr 35	0	0	0	0	0	8.1	0	0	0	11.2	0	0	0	0	2.2	0	0
Fr 36	0	0	0	0	0	10.3	0	0	0	10.8	0	0	0	0	0.7	0	0
Fr 37	0	0	0	0	0	5.9	0	0	0	7.3	0	0	0	0	0	0.3	0
Fr 38	0	0	2.4	0	0	2.8	0	0	0	8.9	0	0	2.6	0	0	0.7	0
Fr 39	0	0	4.6	0	0	2.3	0	0	0	9.8	0	0	3.7	0	0	1.1	0
Fr 40	0	0	9.7	0	0	1.6	0	0	0	9.4	0	0	5.8	0	0	2.4	0
Fr 41	0	0	14.4	4.6	0	1.4	0	0	0	7.3	0	0	8.9	0	0	5.2	0
Fr 42	0	0	19.3	3.9	0	2.3	0	0	0	6.5	0	0	13.4	0	0	11.6	0
Fr 43	0	0	18.0	4.9	0	3.3	0	0	0	4.8	0	0	14.7	0	0	17.4	0
Fr 44	0	0	12.3	9.4	0	4.9	0	0	0	2.8	0	0	12.8	9	0	23.3	0
Fr 45	0	0	5.1	15.5	0	6.9	0	0	0	0	0	0	7.9	18.5	0	32.6	0
Fr 46	0	0	1.7	24.4	0	7.2	0	0	0	0	0	0	4.3	30.4	0	27.7	0
Fr 47	0	0	0	30.1	0	4.4	0	0	0	0	0	0	0	42.3	0	20.2	0

**Fr 48**	0	0	0	23.5	0	2.1	0	0	0	0	0	5.7	0	59.5	0	7.0	0
**Fr 49**	0	0	0	10.9	0	1.0	0	0	12.3	0	4.5	25.9	0	45.4	0	0	0
**Fr 50**	0	0.4	0	6.5	0	0.6	0	0	19.8	0	10.5	31.8	0	28.7	0	0	1.7
**Fr 51**	1.1	0.8	0	0	0	0.7	0	0	22.4	0	23.4	30.4	0	18.2	0	0	3.0
**Fr 52**	2.1	1.5	0	0	0	0.6	0	0	21.3	0	41.0	22.3	0	5.7	0	0	5.5
**Fr 53**	4.0	3.2	0	0	0	0.6	0	0	14.2	0	57.5	12.1	0	0	0	0	8.3
**Fr 54**	7.9	7.1	0	0	2.1	0.6	0	0	5.7	0	59.9	4.4	0	0	0	0	12.3
**Fr 55**	13.0	13.4	0	0	6.9	0.5	0	0	4.3	0	44.9	0	0	0	0	0	17.0
**Fr 56**	16.7	20.5	0	0	19.5	0.6	0	0	2.6	0	20.5	0	0	0	0	0	19.6
**Fr 57**	10.6	16.8	0	0	41.4	0.9	7.9	0	0	0	9.8	0	0	0	0	0	12.6
**Fr 58**	2.8	6.3	0	0	45.0	1.8	39.3	0	0	0	0	0	0	0	0	0	4.8
**Fr 59**	0	0	0	0	10.5	3.6	85.8	0	0	0	0	0	0	0	0	0	0
**Fr 60**	0	0	0	0	0	0.3	8.0	5.4	0	0	0	0	0	0	0	0	0
**Fr 61**	0	0	0	0	0	0	0	8.6	0	0	0	0	0	0	0	0	0
**Fr 62**	0	0	0	0	0	0	0	11.2	0	0	0	0	0	0	0	0	0
**Fr 63**	0	0	0	0	0	0	0	9.9	0	0	0	0	0	0	0	0	0
**Fr 64**	0	0	0	0	0	0	0	5.1	0	0	0	0	0	0	0	0	0
**Fr 65**	0	0	0	0	0	0	0	0	0	0	0	0	0	0	0	0	0
**Fr 66**	0	0	0	0	0	0	0	0	0	0	0	0	0	0	0	0	0
**Fr 67**	0	0	0	0	0	0	0	0	0	0	0	0	0	0	0	0	0
**Fr 68**	0	0	0	0	0	0	0	0	0	0	0	0	0	0	0	0	0
**Fr 69**	0	0	0	0	0	0	0	0	0	0	0	0	0	0	0	0	0
**Fr 70**	0	0	0	0	0	0	0	0	0	0	0	0	0	0	0	0	0

*The blue cells indicate false positive compounds, while the red ones indicate active compounds.

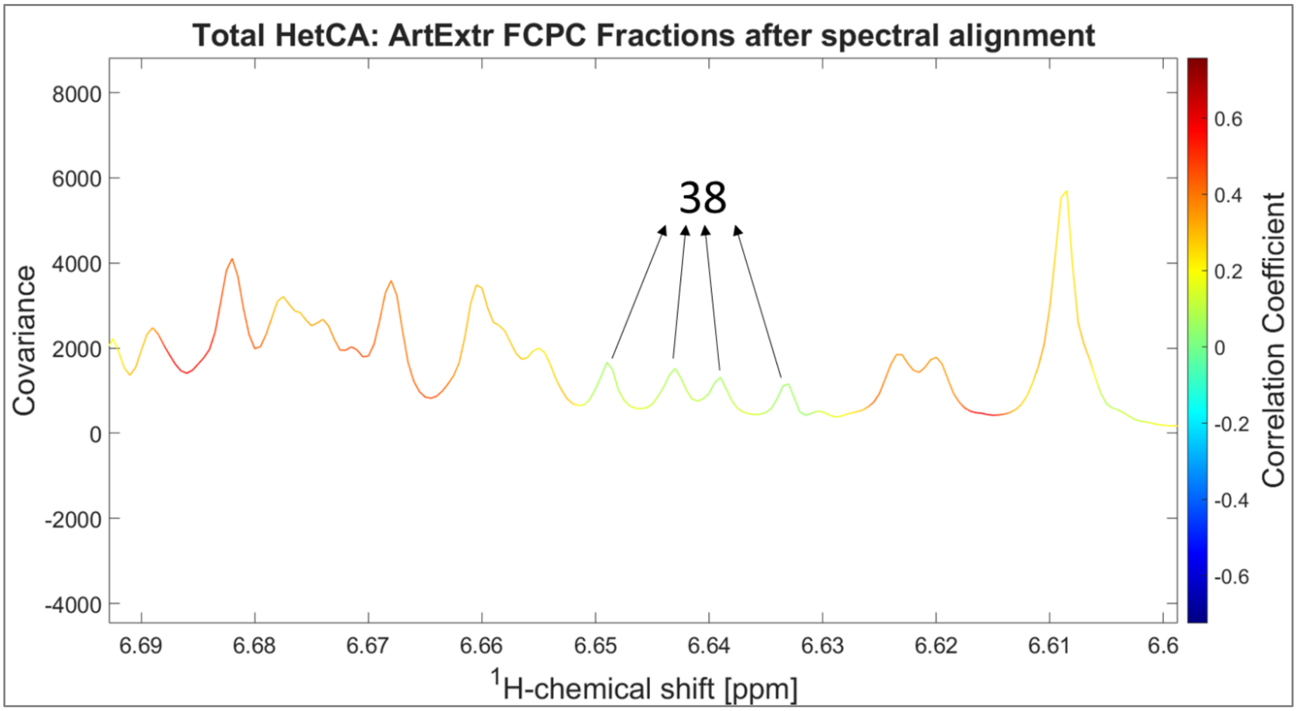

Figure S11. Region of the Total HetCA plot (6.69-6.60 ppm), where one of the peaks of compound **38** (catechol) is displayed.

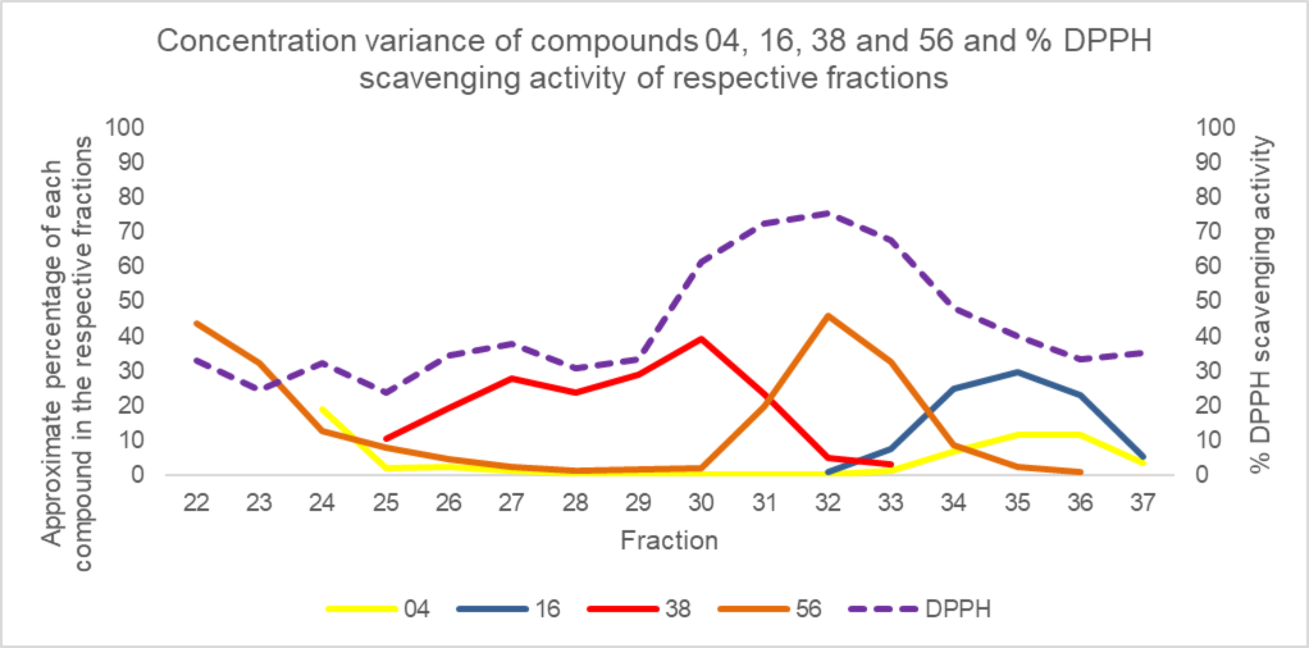

Figure S12. Concentration variance of compounds **04**, **16**, **38** and **56** (kaempferol, hesperetin, catechol and baicalein, respectively) in fractions Fr21-37 and % DPPH scavenging activity of respective fractions.

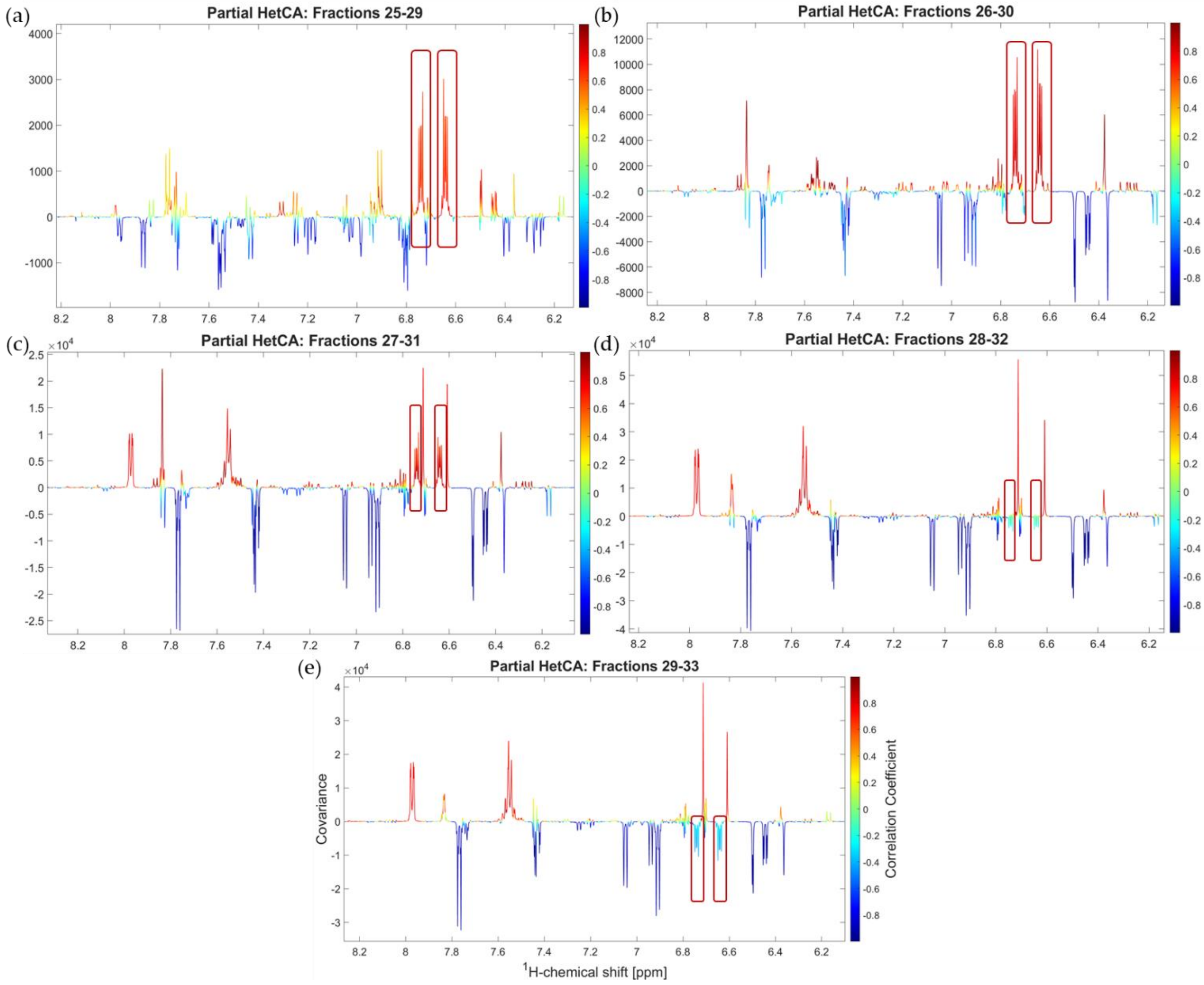

Figure S13. Partial HetCA plots (8.20-6.20 ppm) resulted from the covariance of biological activity against DPPH with corresponding NMR data of fractions (a) Fr25-29, (b) Fr26-30, (c) Fr27-31, (d) Fr28-32 and (e) Fr29-33. The highlighted peaks correspond to **38** (catechol). The left Y axis of each HetCA plot denotes the covariance, the right Y axis represents the correlation coefficient and the X axis indicates the ^{1}H-chemical shift (ppm).

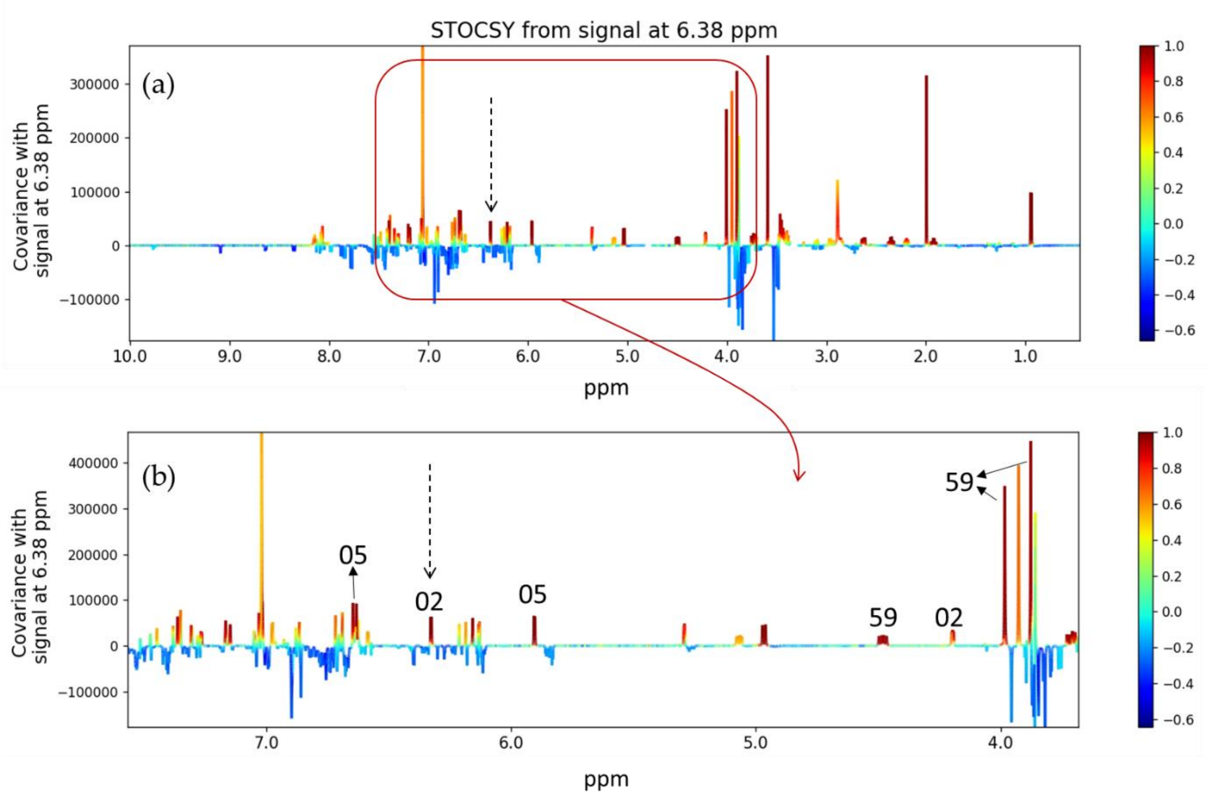

Figure S14. a) Example of STOCSY pseudo-spectrum from signal at 6.38 (compound **02**, quercitrin) ppm in ArtExtr fractions Fr20-60 (10.0-0.8 ppm) and b) zoomed area (7.5-3.8 ppm). The dark red peaks with correlation over 0.94, correspond to compounds **02**, **05** and **59** (quercitrin, phlorizin and colchicine, respectively).

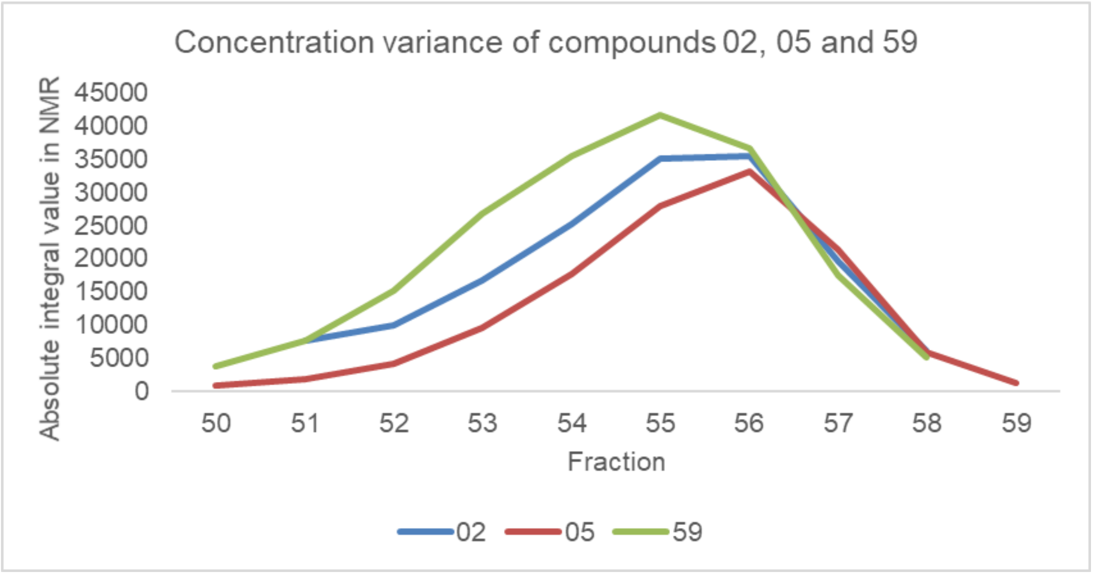

Figure S15. Concentration variance of compounds **02**, **05** and **59** (quercitrin, phlorizin and colchicine, respectively) in fractions Fr50-59.

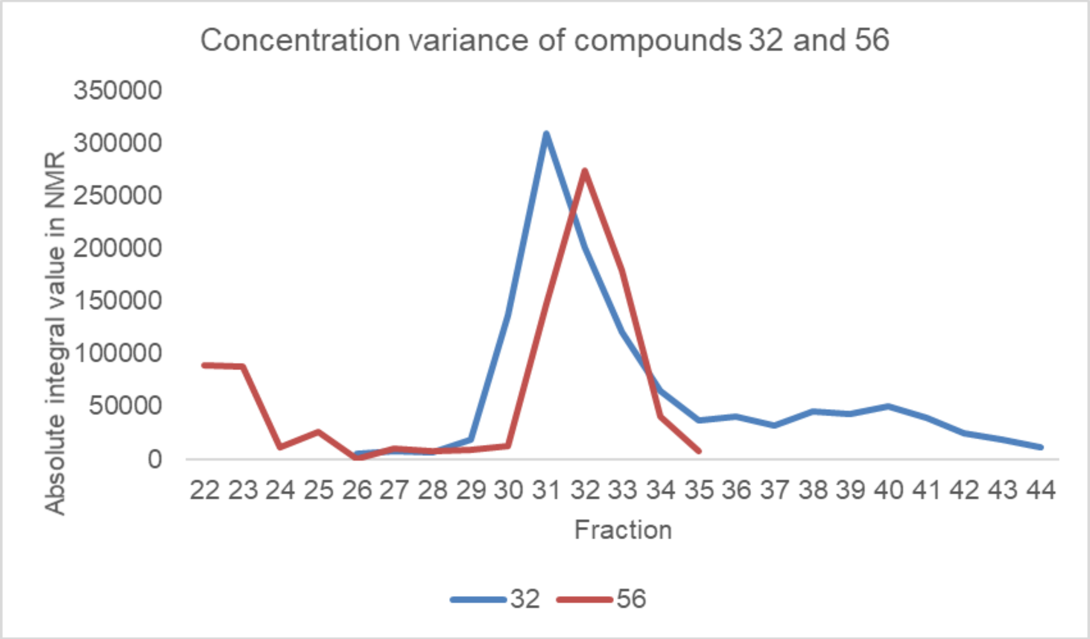

Figure S16. Concentration variance of compounds **32** and **56** (caffeine and baicalein, respectively) in fractions Fr22-44.

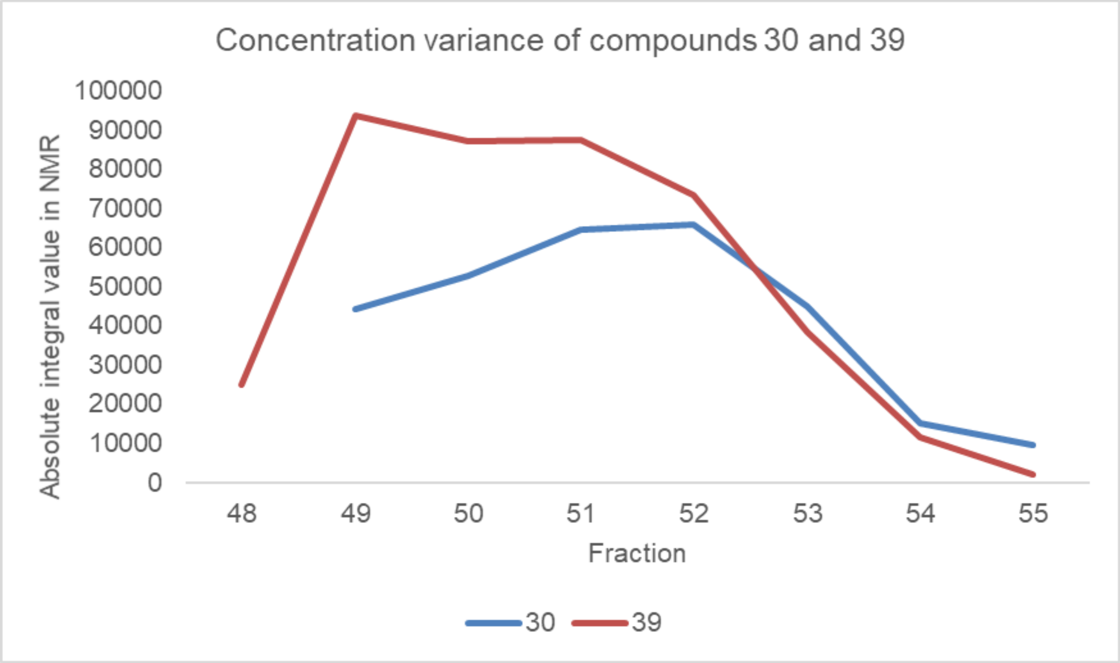

Figure S17. Concentration variance of compounds **30** and **39** (ellagic acid and 3,5-dihydroxybenzoic acid, respectively) in fractions Fr48-55.

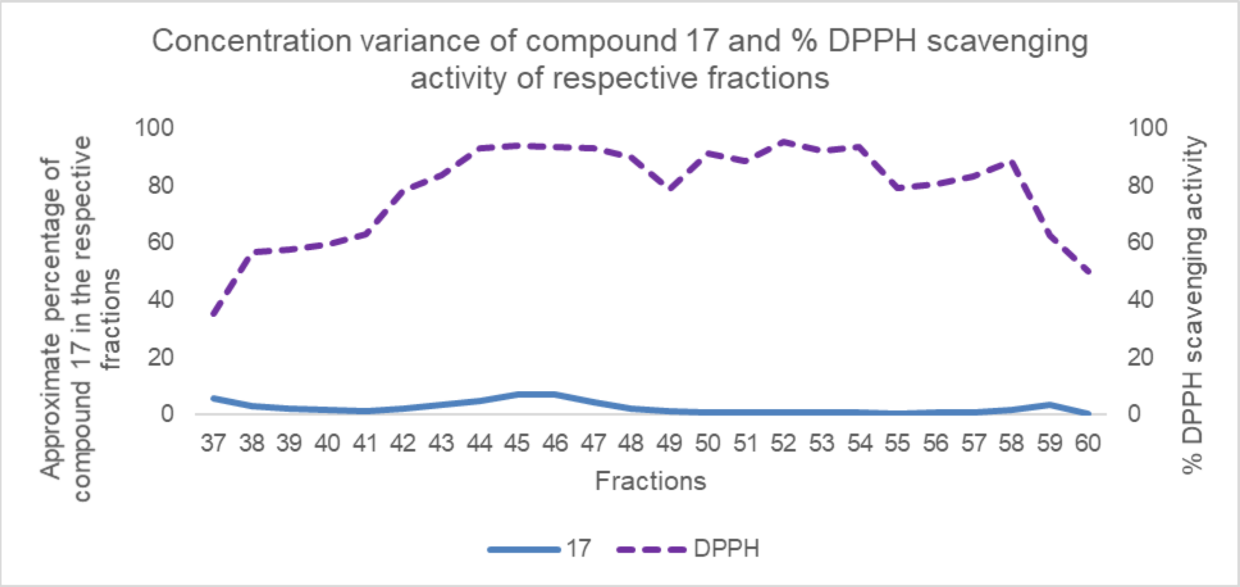

Figure S18. Concentration variance of compound **17** (nicotinic acid) and % DPPH scavenging activity of respective fractions.

Table S8. Results of HetCA in series of five consecutive ArtExtr fractions (Fr20-70) after spectra alignment

Code	Name	Prediction	Code	Name	Prediction
01	Galanthamine hydrobromide	n.i.	31	Tannic acid	n.i.
02	Quercitrin	False negative	32	Caffeine	False positive
03	Quercetin	Ambiguous	33	Vanillin	Correct
04	Kaempferol	False negative	34	Gallic acid	Correct
05	Phlorizin	Correct	35	Biochanin A	Absent
06	Resveratrol	Ambiguous	36	Daidzein	Ambiguous
07	Aristolochic acid	False positive	37	Naringin	Correct
08	Palmitic acid	Absent	38	Catechol	Ambiguous
09	Reserpine	n.i.	39	3,5-dihydroxybenzoic acid	Correct
10	Caffeic acid	Correct	40	D-(−)-quinic acid	n.i.
11	Rosmarinic acid	Ambiguous	41	Ferulic acid	False negative
12	Ephedrine	n.i.	42	4-hydroxybenzoic acid	Correct
13	Harmine	n.i.	43	*m*-Coumaric acid	Correct
14	Oleanolic acid	Absent	44	Isoferulic acid	False positive

15	Naringenin	False positive	45	4-Hydroxybenzaldehyde	Correct
16	Hesperetin	Correct	46	Sinapic acid	Ambiguous
17	Nicotinic acid	Ambiguous	47	Vanillic acid	Correct
18	Shikonin	Absent	48	Diosgenin	Absent
19	Thymol	Absent	49	*p*-Hydroxyphenylacetic acid	False positive
20	Oxytetracycline hydrochloride	n.i.	50	Chlorogenic acid	Correct
21	18-β-glycyrrhetinic acid	Correct	51	Aucuboside	Correct
22	2,4-dimethoxyphenylacetic acid	Correct	52	Sclareol	Absent
23	Curcumin	False negative	53	Protocatechic acid	False negative
24	Oleuropein	Correct	54	(−)-Scopolamine methyl bromide	Correct
25	Sucrose	Correct	55	Loganin	Ambiguous
26	Rutin	Correct	56	Baicalein	Correct
27	Arbutin	False positive	57	6,7-Dihydroxycoumarin (Esculetin)	Correct
28	*p*-Coumaric acid	Correct	58	Umbelliferone	Correct
29	Homovanillic acid	False negative	59	Colchicine	Correct
30	Ellagic acid (dihydrate)	Correct			

n.i.: Not included in the study, Absent: Absent from the fractions 20-70
